# DSC: Containment-Aware Instance Consolidation for Ultra-High-Resolution Railway Signal-Layout Image Analysis

**DOI:** 10.3390/s26206413

**Published:** 2026-10-10

**Authors:** Wenzhen Kuang, Xixuan Tang, Xiao Fu, Wei Xi

**Affiliations:** 1 Institute of Automatic Control, Lanzhou Jiaotong University, Lanzhou 730070, China; kuangwz@lzjtu.edu.cn; 2 Key Laboratory of Optoelectronic Technology and Intelligent Control Ministry of Education, Lanzhou Jiaotong University, Lanzhou 730070, China; 3 National Key Laboratory of Human-Machine Hybrid Augmented Intelligence, National Engineering Research Center for Visual Information and Applications, Xi’an 710049, China; xiaofucn@stu.xjtu.edu.cn (X.F.); xiwei@xjtu.edu.cn (W.X.); 4 School of Computer Science and Technology, Xi’an Jiaotong University, Xi’an 710049, China

**Keywords:** high-resolution image analysis, instance segmentation, tiled inference, duplicate suppression, engineering drawings, railway signal layouts

## Abstract

High-resolution engineering drawings are commonly analyzed in overlapping tiles, which can produce several predictions showing different extents of the same object. Such observations may have low intersection-over-union despite substantial foreground containment. We propose document-space consolidation (DSC), which projects local instance predictions into drawing coordinates and applies class-wise box non-maximum suppression followed by confidence-prioritized mask intersection-over-minimum suppression. This two-stage procedure removes redundant observations while preserving the geometry of retained masks. On ten held-out railway signal-layout drawings containing 1755 annotated instances, the validation-selected configuration achieves precision 0.8994, recall 0.9470, and F1 0.9226. With identical candidates and fixed box-NMS/containment thresholds of 0.50/0.60, DSC reduces false positives from 288 to 186 and improves F1 from 0.8989 to 0.9226 relative to box-based containment filtering. Three-seed local-recognition ablations, a joint threshold analysis, and quantitative error and resource measurements characterize the evaluated pipeline. Optical character recognition and railway-specific rules are assessed separately as application refinement. These results support containment-aware consolidation for producing consistent instance inventories from overlapping tiles in the studied engineering drawings.

## 1. Introduction

Railway signal-layout drawings combine equipment symbols, text, junctions, and long thin track elements. Unlike natural images, engineering drawings contain structured graphical entities whose identity depends on document-level spatial consistency. Recognizing these elements therefore requires both local category and contour estimates and a consistent instance inventory across the drawing. The rasters considered here reach approximately 9624×2648 pixels and are processed with overlapping 768×768 patches.

Direct CAD/DXF parsing depends on layer names, block definitions, and symbol construction; heterogeneous drafting conventions motivate raster-based recognition [1]. Tiling preserves local detail but produces unequal-extent observations: an instance may be complete in one tile and truncated in another. Conventional IoU-based suppression can retain both when their overlap is small relative to their union.

The study separates local recognition, instance consolidation, and application refinement. The adapted local predictor, DSC-Seg, generates class-labelled masks from overlapping tiles. After projection into drawing coordinates, document-space consolidation (DSC) applies box NMS followed by confidence-prioritized mask Intersection-over-Minimum (IoM) suppression. Local training adaptations improve candidate generation; the two-stage operator selects among those candidates without changing retained mask geometry. OCR and railway rules act subsequently as application refinement. Related box-IoS criteria already address containment [2]; DSC evaluates the second-stage containment decision on foreground masks. It cannot reconstruct a complete instance when every available observation is truncated.

The essential distinction is how the two stages handle different overlap patterns. Box NMS first removes competing hypotheses with similar spatial extent; mask-IoM then divides the shared foreground area by the smaller mask area in each same-class pair. This overlap measure is symmetric; confidence priority determines which observation survives, so the retained mask is not necessarily the larger or more complete one. For thin or irregular elements, a bounding rectangle includes substantial background, so box containment need not imply foreground containment. DSC makes the second decision on mask pixels while preserving confidence order and candidate geometry. Neither tiling, NMS, nor containment normalization alone is claimed as new. The contribution is their ordered use for unequal-extent observations, supported by a fixed-candidate comparison that exposes both duplicate reduction and the risk of suppressing distinct instances.

The study focuses on the visual-information analysis layer of railway engineering digitization. Rasterized railway signal-layout drawings are treated as ultra-high-resolution industrial visual inputs, and the resulting instance inventory is represented in the original drawing coordinate system to support engineering review and downstream digital interpretation.

The contributions are:

1. A document-space formulation connecting tiled recognition to a category-aware whole-drawing instance set through consistent projection and evaluation.
2. A deterministic, two-stage selection operator that removes similar-extent box hypotheses before testing containment of the surviving foreground masks, addressing unequal-extent observations without synthesizing mask geometry.
3. A fixed-candidate evaluation separating local recognition from drawing-level consolidation; the controlled comparison shows both the precision gain and the small recall cost of containment-aware selection, while OCR and railway rules are reported only as a separate application refinement.

The study concerns a fixed railway category vocabulary. The application refinement acts on the DSC-core inventory; broader drawing transfer and topology validation remain outside the experiments.

## 2. Related Work

### 2.1. Engineering Drawings, Symbols, and Connectivity

Image analysis for engineering drawings combines region classification, graphical-element recognition, and OCR-oriented interpretation. Prior work on layout parsing and symbol recognition provides useful geometric and semantic foundations [3,4,5,6,7,8].

Electrical-schematic and P&ID studies show that lines and junctions determine the network interpreted from recognized symbols [9,10]. Railway parsers exploit drafting conventions [1], and learned detectors accommodate visual variation [11,12]; box-only outputs, however, do not retain elongated contours. DSC therefore produces masks in drawing coordinates as an input to subsequent interpretation.

### 2.2. Tiled Recognition and Cross-Tile Consolidation

High-resolution slicing creates boundary-truncated observations and requires spatial mask consolidation [13]. Association depends on class, confidence, overlap, and containment because document tiles provide neither temporal continuity nor camera geometry. Box NMS and Soft-NMS [14] can retain unequal-extent duplicates; box-IoS criteria address containment [2], and Weighted Boxes Fusion combines compatible boxes [15]. The operator instead selects existing candidates using box NMS followed by mask-IoM suppression. It does not fuse fragments or infer their physical identity.

Remote SAMsing combines SAM2 candidate generation, context padding, and boundary best-match merging [16]. For comparison, we adapt its merging stage to common railway-labelled predictor outputs. The overlapping-tile adapter uses shared foreground pixels inside source-patch intersections for best-match association; the separate non-overlapping useful-area adapter uses fixed-boundary label merging. Neither comparison uses SAM2-generated masks or the complete Remote SAMsing pipeline. These comparisons concern consolidation operators rather than complete recognition systems. Transformer recognition and geometry-aware supervision support the local predictor [17,18,19]; their ablations are evaluated separately from fixed-candidate consolidation.

### 2.3. Challenges in Containment-Aware Consolidation

Tile duplication is not a single geometric situation. A complete instance may coexist with a truncated boundary fragment, the same instance may be observed at different extents in two overlapping tiles, and two neighboring instances of the same class may genuinely overlap. In the first two cases, the smaller observation is often substantially contained in the larger one. Union-normalized IoU can remain modest when containment is strong, whereas IoM measures the fraction of the smaller support explained by the larger support. The operator therefore uses confidence-prioritized containment suppression for unequal-extent observations: box NMS removes near-identical hypotheses first, and mask-IoM then tests support inclusion on the survivors. This formulation does not assume that every containment relation is a duplicate; independent neighboring instances remain a documented suppression risk.

### 2.4. Intelligent Analysis Across Imaging and Monitoring Domains

High-resolution recognition is also central to remote sensing and semiconductor inspection. Adaptive slicing in remote-sensing images allocates local processing to preserve small-object information [20]; slicing with refinement has also been studied for small defects in scanning-electron-microscope images of advanced integrated-circuit patterns [21]. These studies motivate retaining local detail, but the present problem additionally requires selecting among overlapping masks that depict different extents of the same engineering element. DSC addresses that output-reconciliation step rather than the choice of slicing strategy alone.

Recent infrastructure research provides a broader reliability context. Kernel-regression-enhanced Bayesian dynamic linear modeling supports prediction and missing-data imputation for structural-monitoring streams under typhoon conditions [22], while Bayesian-network research reviews probabilistic reasoning and multisource information fusion for structural health monitoring [23]. Rail-corrugation research connects physical formation mechanisms with detection methods [24]; Wang et al. use axle-box vibration preprocessing and a CNN–LSTM model for on-board corrugation diagnosis [25]. These monitoring studies concern temporal signals and physical condition assessment, not cross-tile instance segmentation. Their relevance here is the need to state uncertainty and domain limits explicitly; DSC does not infer structural condition or replace sensor-based monitoring.

Accordingly, the closest methodological comparisons are spatial consolidation operators. Box-IoS filters compare enclosing rectangles; SMM groups candidates and unions their masks; the SAM-Mosaic adapters associate cross-tile observations by foreground or boundary contact. DSC instead retains a confidence-prioritized subset after ordered box and mask tests. This distinction matters when the available observations differ in extent and when mask union could join neighboring elements. It also explains a limitation: disjoint fragments provide no containment evidence and cannot be reconnected by DSC.

### 2.5. Document-Level Reliability

Overlapping patches share content, so complete drawings are the resampling units for core uncertainty intervals. Training-seed dispersion, overlap sensitivity, and pixel-domain localization residuals describe distinct sources of variation and are reported separately.

## 3. Proposed Framework

**Figure 1:**
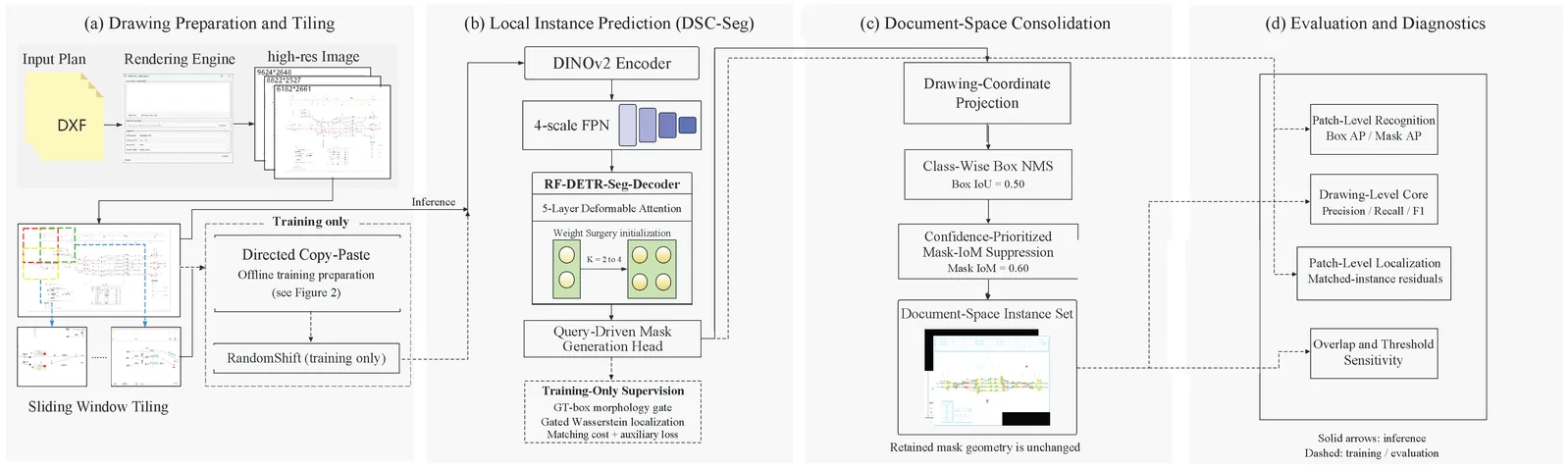
Local prediction, document-space consolidation, and evaluation. Training-only augmentation applies directed Copy–Paste followed by RandomShift. Weight Surgery initializes expanded attention sampling; gated Wasserstein localization provides training supervision. At inference, DSC-Seg predicts tile instances, which are projected into drawing coordinates and filtered by box NMS followed by confidence-prioritized mask-IoM suppression. Retained mask geometry is unchanged. Dashed branches distinguish training and evaluation from the solid inference path. Patch-level AP and matched-instance localization diagnostics are evaluated before consolidation; drawing-level core metrics and sensitivity analyses evaluate the resulting instance inventory. OCR and railway rules are assessed separately as subsequent application refinement.

Figure 1 separates training, inference, and evaluation for local recognition and DSC-core consolidation. The local predictor $h_{\theta }$ (DSC-Seg) is evaluated by patch AP. The DSC core, $g_{\tau }\circ T\circ h_{\theta }$, projects local predictions with *T* and applies the two-stage consolidation operator $g_{\tau }$; its output is evaluated by drawing-level instance precision, recall, and F1. Application refinement *r* adds OCR and railway rules, producing $r(g_{\tau }\left(T\left(h_{\theta }\left(P\right)\right)\right))$. Here $h_{\theta }\left(P\right)$ denotes prediction over all tiles. Refinement does not rerun the predictor or consolidation. Fixed-candidate comparisons isolate $g_{\tau }$, whereas local-recognition ablations assess $h_{\theta }$; improvements in refined-output F1 are attributed to the additional refinement stage.

### 3.1. Problem Formulation and Observation Model

Let *I* be a rasterized drawing and $P={\left\{\left(P_{j},o_{j}\right)\right\}}_{j=1}^{J}$ its overlapping tiles and top-left offsets. Candidate generation and consolidation are

(1) $${\hat{Y}}_{j}=h_{\theta }\left(P_{j}\right),\;\hat{I}=g_{\tau }\;\left(\overset{J}{\underset{j=1}{⋃}}T_{o_{j}}\left({\hat{Y}}_{j}\right)\right).$$

The union denotes concatenation with candidate identity retained. Each candidate contains a class, confidence, box, binary mask, tile ID, and offset. The final core output is a set of retained instances in drawing coordinates; geometric attributes such as centroids are derived from their masks.

For $o_{j}=\left(o_{j}^{x},o_{j}^{y}\right)$, a local box $\left(x_{1},y_{1},x_{2},y_{2}\right)$ is translated to $\left(x_{1}+o_{j}^{x},y_{1}+o_{j}^{y},x_{2}+o_{j}^{x},y_{2}+o_{j}^{y}\right)$. The binary mask is placed on the drawing pixel grid at the same offset. Boxes and masks are clipped to drawing bounds; empty masks are discarded. Tile IDs and offsets support projection and record keeping. DSC consolidation uses class, confidence, boxes, and masks for suppression, with original candidate order resolving score ties.

Source CAD drawings are rasterized with a diagram-specific DPI to retain fine symbols. Pixel-to-CAD scale is therefore not constant across drawings. All reported localization residuals are pixel-domain diagnostics, not physical measurement errors.

### 3.2. Local Graphical-Element Recognition

An RF-DETR-Seg-Large-based model [18] generates the local candidates. Its instance-mask output and deformable-attention decoder support the sampling-point expansion evaluated here. Section 4.3 provides a descriptive comparison with YOLO11-seg-L [26], YOLO26-seg-L [27], and Mask2Former [28]; the controlled component comparisons appear in the same section. The local adaptations address rare-class coverage, elongated-object sampling, input-grid alignment, and compact-target matching. DSC consolidation accepts projected class-labelled masks, boxes, and scores and is not tied to this architecture, although its effect with other candidate generators is not evaluated here. The Weight Surgery numerical check appears in Supplementary Section S-III.

#### 3.2.1. Data-Level Compensation: Directed Copy–Paste

**Figure 2:**
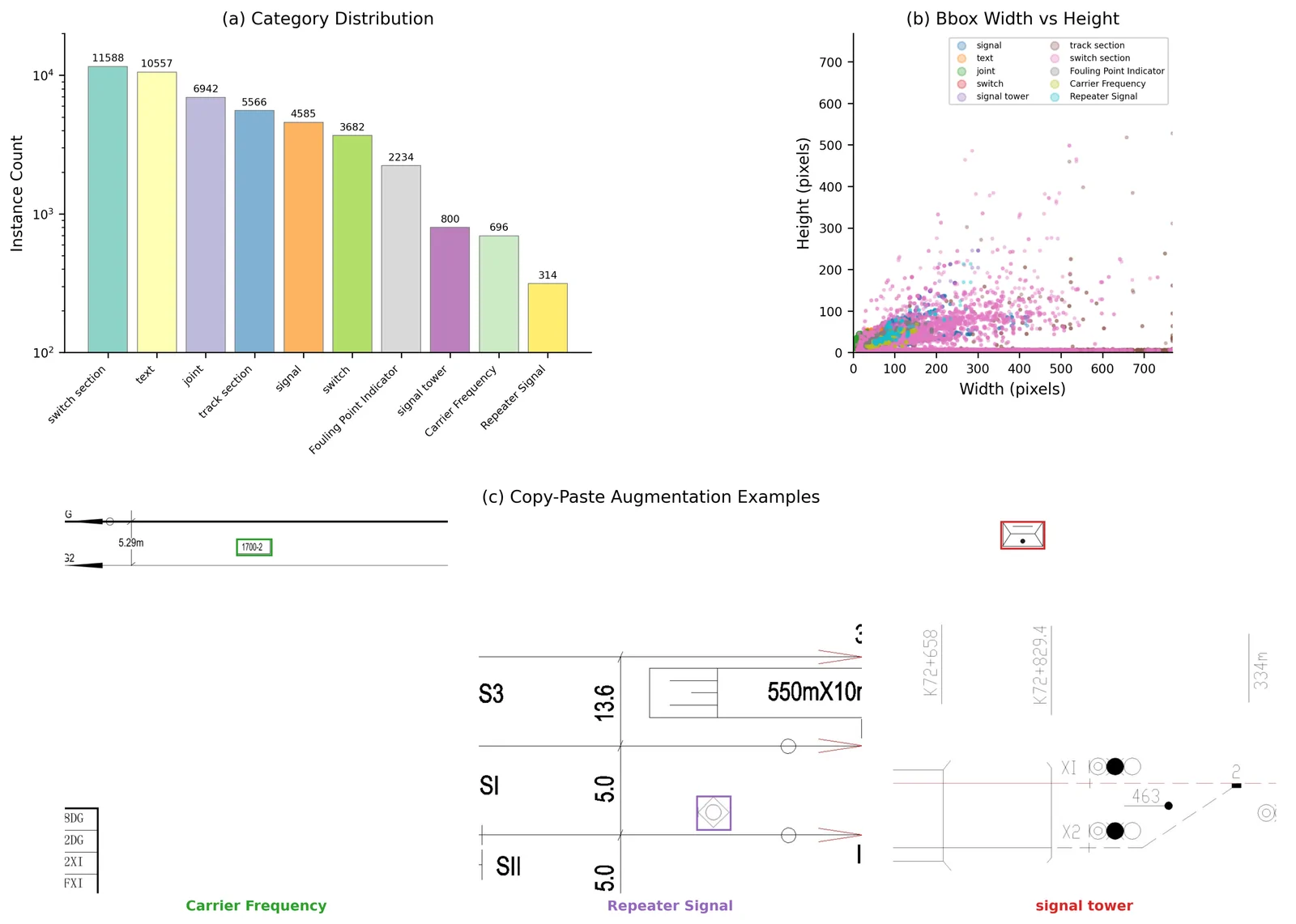
Dataset statistics and training-only Copy–Paste examples. The category-count and bounding-box-size panels summarize the original annotations across all complete source drawings before augmentation and exclude pasted instances. The augmentation examples illustrate Copy–Paste applied only to training patches; they are separate from the population used for the statistical panels.

The pre-augmentation class-frequency statistics across all 81 complete source drawings exhibit a pronounced long-tailed distribution. Signal tower, Repeater Signal, and Carrier Frequency are the three least frequent categories in these dataset-level statistics and are therefore selected for directed Copy–Paste [29]. This category selection is a dataset-design decision based on pre-augmentation class counts; the augmentation operation itself remains training-only. Complete instances are inserted into compatible training-background regions under non-overlap and intensity-consistency checks. Figure 2 includes training-only Copy–Paste examples. In the current training export, 570 blank training patches are modified in place with 780 added instances: 380 signal towers, 300 Repeater Signals, and 100 Carrier Frequency instances. The final image count is unchanged; Table 1 gives the counts and source restrictions. Pasted instances and background patches are drawn only from the training split; no validation or test image is used as augmentation material.

#### 3.2.2. Representation-Level Compensation

*Sampling support and initialization.* Deformable-attention sampling expands from K=2 to K=4 to provide additional support for elongated structures. Weight Surgery duplicates pretrained offsets and attention logits, scaling the raw offsets by $K^{\prime }/K$ to compensate for the implementation’s sampling-count normalization. The initialized response is equivalent before symmetry-breaking perturbation; the construction and proof follow below. A numerical implementation check is retained in the Supplementary Material, Section S-III.

**Proposition** **1.** *Expanding box-conditioned deformable attention from K to $K^{\prime }=2K$ preserves its output before perturbation when raw offsets are duplicated and scaled by $K^{\prime }/K$, and attention logits are duplicated without scaling.*

**Proof.**  For one head and level, the original sampling position and output are

(2) $$x_{k}=x_{\mathrm{ref}}+\frac{\Delta x_{k}}{K}s_{\mathrm{ref}}\;0.5,\;y=\sum_{k=1}^{K}p_{k}V\left(x_{k}\right),$$

where $\Delta x_{k}$ is the offset projection and $p_{k}=\mathrm{softmax}{\left(z\right)}_{k}$. Within each head/level output block, set the offset-projection weights and biases (subscript s) and attention-logit weights and biases (subscript a) as

(3) $$\begin{array}{cccc} W_{s}^{\prime } & =2[W_{s};W_{s}], & b_{s}^{\prime } & =2[b_{s};b_{s}],\\ W_{a}^{\prime } & =[W_{a};W_{a}], & b_{a}^{\prime } & =[b_{a};b_{a}]. \end{array}$$

The duplicated offsets satisfy $\Delta x_{k}^{\prime }/K^{\prime }=2\Delta x_{k}/\left(2K\right)$, so both copies sample $x_{k}$. Duplicating logits doubles the softmax denominator, giving each copy weight $p_{k}/2$. Hence

(4) $$y^{\prime }=\sum_{k=1}^{K}\left(\frac{p_{k}}{2}+\frac{p_{k}}{2}\right)V\left(x_{k}\right)=y.$$

For multi-level attention, every level’s points must be duplicated: the joint level–point softmax denominator then also doubles. The construction applies per head, preserving the subsequent shared output projection. It requires the box-conditioned reference-point branch with an explicit 1/K offset normalizer; it is not a universal initialization rule for other sampling formulas. In DSC, K=2 and $K^{\prime }=4$.   □

After initialization, Gaussian perturbations are added to the duplicated offset parameters ($\sigma_{s}=0.02$) and attention-logit projection parameters ($\sigma_{a}=0.01$) to break symmetry. Exact equivalence applies before these perturbations.

*Tile-origin augmentation.* RandomShift translates training content by

(5) $$\Delta x,\Delta y\overset{i.i.d.}{∼}\mathrm{Unif}\left\{-12,-11,\ldots ,12\right\}.$$

The encoder uses a 12-pixel sampling lattice, so this range covers every residue class modulo its stride, with residue zero occurring three times and each other residue twice. Exposed regions receive gray value 127 before normalization, and annotations are clipped. This augmentation varies input-grid alignment and boundary position without implying complete phase invariance.

#### 3.2.3. Supporting Supervision Adaptation: Gated Wasserstein Localization

Inspired by normalized Wasserstein distance (NWD) [19], a squared-Wasserstein localization term supplements the standard losses for compact targets. The GT-box gate requires max(w/h,h/w)<7 and normalized GT-box area below 0.04; thresholds were selected using a predefined coarse validation grid, without joint optimization. For normalized center and size coordinates $\left(c_{x},c_{y},w,h\right)$, with subscripts p and g denoting prediction and ground truth, respectively,

(6) $$\begin{array}{cc} W_{2}^{2}= & {\left(c_{x,p}-c_{x,g}\right)}^{2}+{\left(c_{y,p}-c_{y,g}\right)}^{2}\\ \; & +\frac{1}{4}\left[{\left(w_{p}-w_{g}\right)}^{2}+{\left(h_{p}-h_{g}\right)}^{2}\right], \end{array}$$

and $S_{\mathrm{sqW}}=\exp\left(-W_{2}^{2}/C\right)$ with C=0.1. The per-instance auxiliary loss is $L_{\mathrm{sqW}}=1-S_{\mathrm{sqW}}$; $L_{\mathrm{gw}}$ in the total loss denotes its gate-masked mean over eligible matched targets, with zero contribution when no target passes the gate. The regression coefficient is 1.0; Hungarian matching adds $2(1-S_{\mathrm{sqW}})$ for eligible GT targets. The same similarity and GT-box morphology gate are used in the regression loss and matching cost. We refer to this term as the gated Wasserstein localization loss; it is defined directly by the squared-distance expression above rather than by the classical square-root NWD normalization. Standard box and mask terms remain active:

(7) $$\begin{array}{cc} L= & \lambda_{\mathrm{cls}}L_{\mathrm{cls}}+\lambda_{L1}L_{L1}+\lambda_{\mathrm{GIoU}}L_{\mathrm{GIoU}}\\ \; & +\lambda_{\mathrm{gw}}L_{\mathrm{gw}}+\lambda_{\mathrm{mask}}^{\mathrm{ce}}L_{\mathrm{mask}}^{\mathrm{ce}}+\lambda_{\mathrm{mask}}^{\mathrm{dice}}L_{\mathrm{mask}}^{\mathrm{dice}}. \end{array}$$

The gate excludes highly elongated targets from this auxiliary term. Its localization diagnostics are conditional on each configuration’s matched set.

### 3.3. Containment-Aware Candidate Consolidation

The operator consolidates confidence-filtered candidates after the projection in Section 3.1. Candidates are ordered by descending confidence, breaking exact-score ties by ascending original candidate index. Within each class, greedy box NMS first removes candidates with box IoU above $\tau_{\mathrm{IoU}}$ against an already retained candidate. The survivors then undergo a second greedy scan in the same order: candidate *j* is suppressed if a previously retained same-class candidate *i* satisfies

(8) $$\mathrm{IoM}\left(m_{i},m_{j}\right)=\frac{|m_{i}\cap m_{j}|}{\min(|m_{i}|,|m_{j}|)}>\tau_{\mathrm{IoM}}.$$

IoM is symmetric; suppression direction comes from priority. The purpose is not to estimate object similarity, but to detect whether one observation is geometrically explained by another. No cross-class suppression is performed.

*Geometric operating range.* For smaller mask *F* and larger mask *C*, let r=|F|/|C|≤1 and ρ=|F∩C|/|F|. Then

(9) $${\mathrm{IoU}}_{\mathrm{mask}}\left(F,C\right)=\frac{\rho r}{1+r-\rho r},\;\mathrm{IoM}\left(F,C\right)=\rho .$$

Complete containment gives mask IoU *r* but IoM one, explaining why the second stage can suppress unequal-extent candidates. This relation concerns masks; survival of the first stage depends separately on box IoU. IoM does not identify the physical cause of overlap: the operator uses no tile adjacency, boundary-contact, or completeness test. A high-confidence incomplete candidate can suppress a more complete one, and overlapping independent same-class instances remain at risk. The IoM threshold is selected on validation data through a trade-off between recall and false discovery rate (FDR).

*Output properties.* Retained masks are copied unchanged, so DSC consolidation selects observations without fusing masks or reconstructing missing geometry. For fixed thresholds and ordering, its output has no same-class pair exceeding either suppression threshold.

Algorithm 1 summarizes the core; complete projection and suppression pseudocode is provided in the Supplementary Material, Section S-XV.
**Algorithm 1** DSC core: tiled prediction and document-space consolidation**Require:** Raster *I*; tile size 768; stride 614; score cutoff $\tau_{s}=0.20$; $\tau_{b}=\tau_{\mathrm{IoU}}=0.5$; $\tau_{c}=\tau_{\mathrm{IoM}}=0.6$
**Ensure:** Retained document-space instances $\hat{I}$
 1:D←[]; $\hat{I}\leftarrow \left[\;\right]$ 2:**for** tile $\left(P_{j},o_{j}\right)$ under boundary-aligned tiling **do** 3:      Predict $h_{\theta }\left(P_{j}\right)$; keep scores $s\geq \tau_{s}$ 4:      Project boxes and binary masks by $o_{j}$; clip to *I*; discard empty masks; append to D with original indices 5:**end for** 6:**for** each class group in D **do** 7:      Order by descending score, then ascending original index 8:      Apply greedy box NMS at $\tau_{b}$; preserve order 9:      Scan survivors greedily; keep a candidate iff its mask IoM with every previously kept candidate is $\leq \tau_{c}$10:      Append kept candidates unchanged to $\hat{I}$11:**end for**12:**return **$\hat{I}$


#### Application Refinement and Refined Output

The application refinement applies OCR validation and relabeling, followed by railway-specific section-connectivity rules, to the consolidated instances. The DSC core and refined output are evaluated as different stages. Detailed enabled rules are specified in the Supplementary Material, Section S-XIII.

## 4. Experiments

### 4.1. Experimental Setup

#### 4.1.1. Dataset

The Railway Signal Diagram Dataset (RSDD) contains 81 complete railway station-yard signal-layout drawings from four railway bureau group companies and seven signalling maintenance sections. The source-diagram split comprises 57 training, 14 validation, and ten held-out test drawings. No drawing or patch derived from it appears in multiple splits. Copy–Paste is restricted to training. The validation set is used for model selection, threshold calibration, overlap selection, and rule development.

The training and validation pool covers three bureaus and five maintenance sections. Six test drawings come from represented sources; four come from an additional bureau and two additional maintenance sections. The ten test drawings contain 1755 annotated instances. The four unseen-source drawings provide a preliminary generalization diagnostic rather than a broad cross-organization evaluation.

**Figure 3:**
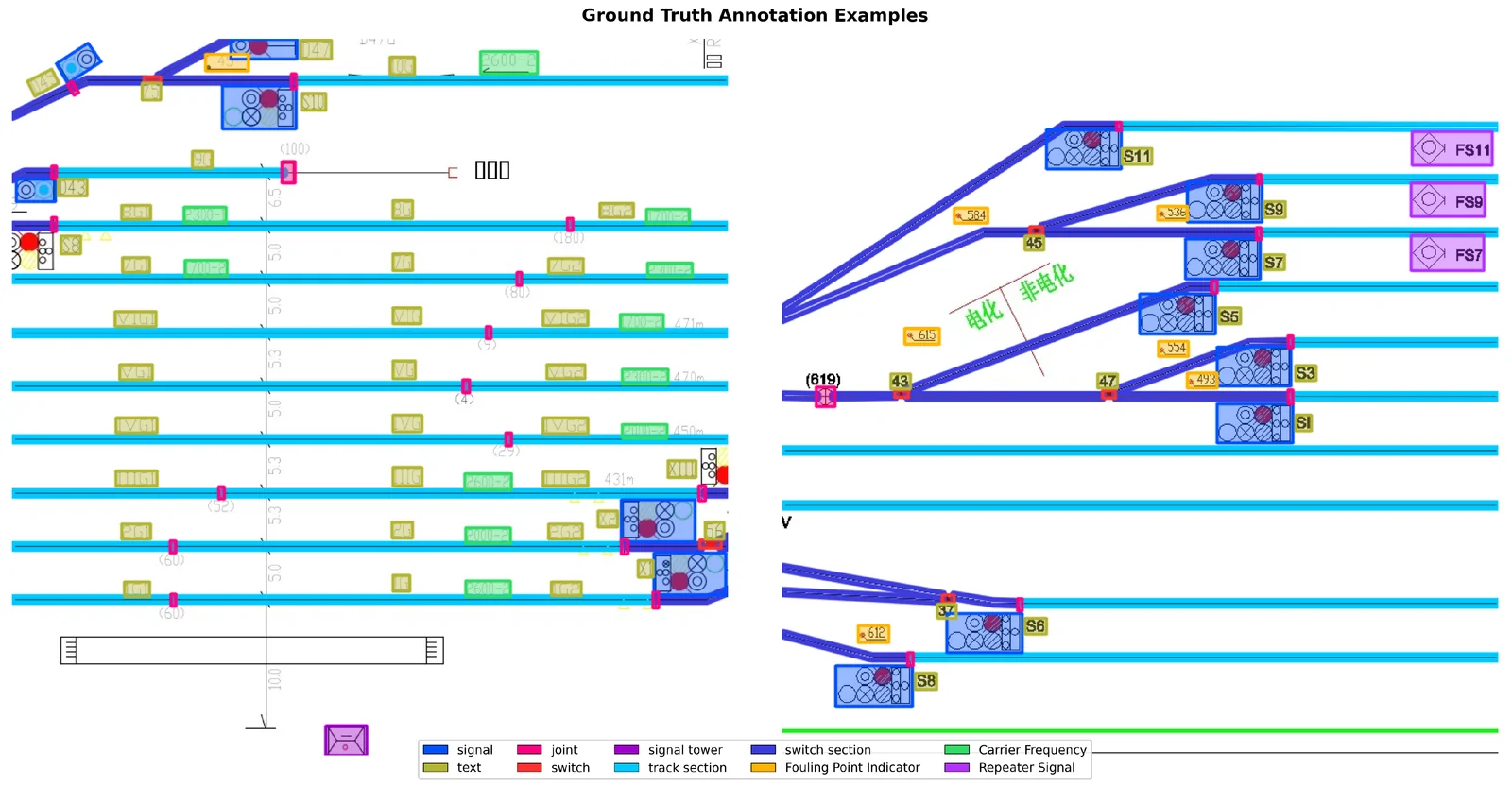
Ground-truth annotation examples from two railway signal-layout regions. The ten categories include signal, text, joint, switch, signal tower, track section, switch section, Fouling Point Indicator, Carrier Frequency, and Repeater Signal. Colors follow the original class legend.

Annotations are produced on full-resolution rasters. Linear targets use narrow polygonal masks following visible centerlines, with approximately 2-pixel margins on each side; text groups use tight rectangular masks. Overlapping instances are annotated independently. All ten test drawings underwent independent review by a railway-signalling-trained annotator. Five instance-level errors across three drawings were corrected in the annotation files used for the reported evaluation. All reported experiments use the corrected annotation version. Figure 3 illustrates the annotation categories. A trained graduate researcher produced the annotations; only the corrected annotation files remain, so the audit is a correction log rather than a recoverable before/after release.

RSDD contains frequent classes, rare equipment categories, elongated track elements, and compact joint symbols. The statistical panels in Figure 2 summarize the original annotations across all complete source drawings before augmentation; the same figure also contains training-only Copy–Paste examples. Counts range from 11,588 switch-section instances to 314 Repeater Signal instances (approximately 36.9:1), demonstrating a long-tailed class distribution. Signal tower, Repeater Signal, and Carrier Frequency are the three least frequent categories in these whole-dataset pre-augmentation statistics and are the categories targeted by directed Copy–Paste. These whole-dataset counts exclude pasted instances and are distinct from the corrected held-out-test counts reported below in the DSC-core results. Table 1 reports the training export after offline augmentation; modified blank images replace their originals.

Training annotations are generated by intersecting each full-drawing polygon with the patch rectangle. Non-line categories are retained when intersection-over-foreground (IoF), defined as intersection area divided by original instance area, meets the category threshold: text 1.0, joint 0.9, signal/switch 0.7, signal tower 0.6, and Fouling Point Indicator/Carrier Frequency/Repeater Signal 0.7. Track-section and switch-section fragments are retained when clipped area is at least 60 pixels^2^. The intersected polygon is translated to patch coordinates and clipped to the patch boundary; its bounding box is recomputed from the clipped geometry. Empty intersections are discarded; non-line instances failing their category-specific IoF threshold and line fragments below the 60-pixel^2^ threshold are excluded. This training-time rule is separate from evaluation, where no additional 5% visible-area ignore rule is applied.

#### 4.1.2. Evaluation Metrics and Protocols

Two evaluation spaces answer complementary questions. Patch-level COCO-style Box/Mask AP measures local recognition before document-space consolidation. Drawing-level precision, recall, false discovery rate (FDR), and F1 measure the projected and consolidated instance output; drawing-level matching and localization residuals use class-aware mask IoU ≥0.5. Local AP is evaluated over the confidence-ranked predictions, without the fixed 0.20 pre-filter used for drawing-level inference. The refined output is evaluated separately from the DSC core.

For each complete drawing, predictions and GT instances are grouped by class. Predictions are processed in descending confidence order and greedily matched to the unmatched same-class GT with maximum mask IoU, provided IoU is at least 0.5. Original candidate order resolves score ties and GT index resolves equal-IoU ties. Unmatched predictions are FP and unmatched GT instances are FN. No patch-visible-area exclusion is applied to complete-drawing evaluation.

(10) $$\begin{array}{cc} P & =\frac{\mathrm{TP}}{\mathrm{TP}+\mathrm{FP}},\;R=\frac{\mathrm{TP}}{\mathrm{TP}+\mathrm{FN}},\\ \mathrm{FDR} & =\frac{\mathrm{FP}}{\mathrm{TP}+\mathrm{FP}},\;F1=\frac{2\mathrm{TP}}{2\mathrm{TP}+\mathrm{FP}+\mathrm{FN}}. \end{array}$$

Micro metrics are recomputed from pooled counts; drawing-level Macro metrics are arithmetic means of per-drawing values. Class-wise macro averages reported below are explicitly distinguished from drawing-level Macro results. FDR equals 1−P under the same aggregation and zero-denominator convention. Metrics are computed before rounding.

The patch-evaluation split contains 1228 validation patches: 788 with annotations and 440 negative patches. Patches are 768×768 pixels with stride 384 (50% overlap). Right and bottom terminal patches are shifted inward to cover the drawing boundary; drawings smaller than 768 pixels in either dimension are white-padded on the uncovered right/bottom side. The COCO evaluator uses maxDets = 500; because RF-DETR exports at most 200 query predictions per patch (num_select = 200), this evaluator cap is non-binding. Box AP and Mask AP use their respective IoU thresholds from 0.50 to 0.95 in increments of 0.05; AP_50_ and AP_75_ use 0.50 and 0.75. No separate 5% visible-area ignore rule is applied. Further evaluator settings are recorded in the Supplementary Material, Section S-VI.

#### 4.1.3. Implementation Details

Experiments use PyTorch 2.4 and CUDA 12.1 on an NVIDIA RTX 4090.

The RF-DETR-based configurations use 768×768 inputs, 200 queries, batch size 4 with 7× gradient accumulation (effective batch 28), encoder learning rate $5\times 10^{-5}$, two warm-up epochs, decay at epoch 55, and at most 100 epochs. Training uses mixed precision and early stopping with patience 20. Repeated configurations use seeds 2026, 2027, and 2028; the primary frozen core result uses seed 2026 and checkpoint_best_ema.pth, selected by the highest validation Mask AP; the same checkpoint and candidate export are used for the fixed-candidate consolidation comparison. The result registry and reported evaluation settings appear in the Supplementary Material, Section S-VI.

Drawing-level inference uses confidence threshold 0.20, box-NMS IoU threshold 0.5, and mask-IoM threshold 0.6. Nominal overlaps of 0%, 20%, 25%, and 50% were compared exclusively on the validation drawings, holding the checkpoint, confidence cutoff, consolidation parameters, class vocabulary, and drawing-level evaluator fixed; only tile overlap was varied. Selection used drawing-level DSC-core Micro F1 after confidence filtering and DSC consolidation, with exact ties resolved by higher recall and then lower FDR. The selected 20% overlap uses 768-pixel tiles and stride 614, giving a realized regular-grid overlap of 1−614/768≈20.05%. The overlap, stride, and consolidation parameters were then frozen for subsequent method comparisons and held-out evaluation. Terminal tile origins are shifted inward and deduplicated to cover the right and bottom boundaries. Test drawings were not used for overlap selection or parameter tuning. A post-selection sensitivity analysis was additionally performed on the held-out test drawings across the same four overlaps to quantify robustness, with the checkpoint and consolidation parameters held fixed; this analysis did not alter the validation-selected 20% operating point.

Optimization uses AdamW with weight decay $10^{-4}$, encoder learning rate $5\times 10^{-5}$, and decoder/mask-head learning rate $10^{-4}$. The learning-rate decay at epoch 55 has factor 0.1; gradient clipping uses maximum norm 0.1. The starting weights are recorded as an official RF-DETR-Seg-Large checkpoint; the study configuration uses a 12-pixel backbone patch-embedding stride. The exact release identifier and checkpoint hash were not retained. Python 3.10 and Ubuntu 22.04 complete the reported software environment. Table 2 records the key operating values and where they are determined. The validation drawings are used for model selection, overlap selection, threshold calibration, and rule development; held-out test drawings are reserved for final reporting and post-selection sensitivity analysis.

### 4.2. Drawing-Level Core Results and Application Refinement

#### 4.2.1. DSC Core

**Figure 4:**
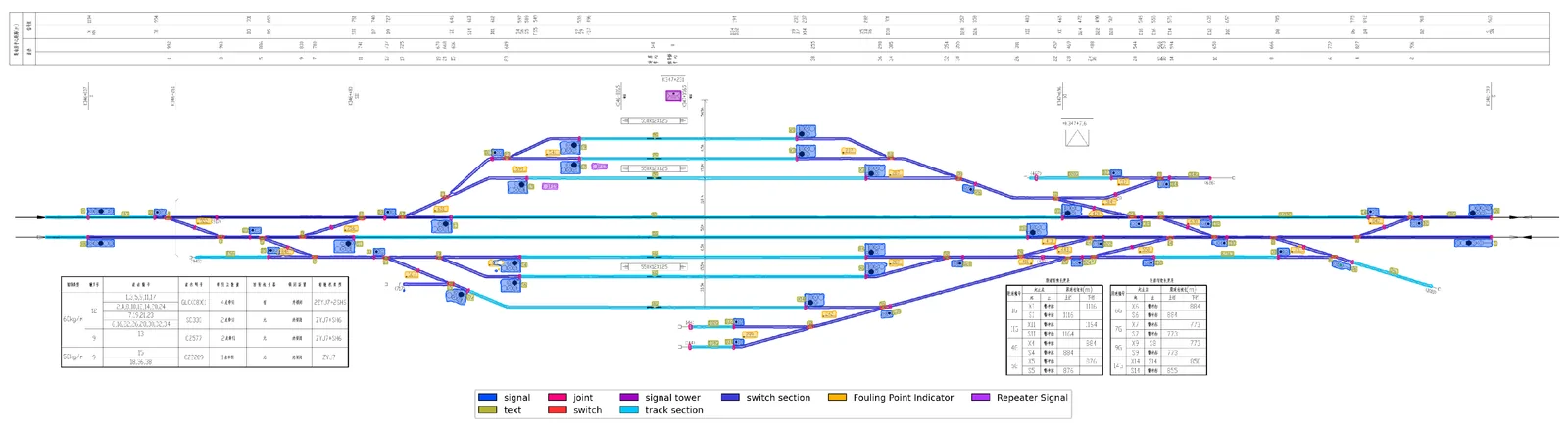
Complete railway signal-layout drawing with DSC-core instance-segmentation output after coordinate projection, class-wise box NMS, and mask-IoM suppression. The full drawing extent is shown; colors identify predicted categories. OCR and railway-specific rule refinement are not applied.

The core output includes local recognition, coordinate projection, class-wise box NMS, and mask-IoM suppression. Across the ten held-out drawings, the pooled counts are TP = 1662, FP = 186, and FN = 93, with 1848 predictions. Table 3 reports the resulting Micro and drawing-level Macro metrics. Table 4 gives class-wise results; per-drawing and source-stratified results are retained in the Supplementary Material, Section S-XII. Figure 4 shows a complete drawing after projection and two-stage consolidation, before OCR or railway-rule refinement. It complements the pooled metrics with a view of the drawing-wide instance inventory.

Carrier Frequency has the lowest class-wise F1 (0.8000), with eight TP, two FP, and two FN among ten GT instances. Signal tower also has only ten GT instances, despite its observed recall of 1.0000. These small denominators limit class-specific generalization claims. The class-wise Macro F1 of 0.9130 weights categories equally and is distinct from drawing-level Macro F1 of 0.9238.

#### 4.2.2. Fixed-Candidate Consolidation

This experiment changes the consolidation operator while keeping the local predictor, pre-consolidation candidate export, confidence cutoff, class labels, initial candidate ordering, and evaluator fixed. One projected candidate list is supplied before every compared operator. Each adapter then applies its own score aggregation and selection rule: DSC consolidation preserves confidence priority, whereas SMM and SAM-Mosaic generate their respective output scores during adaptation. DSC consolidation, the SMM adapter [13], and the SAM-Mosaic overlap-contact adapter [16] process this list under the selected 20% overlap protocol. The SAM-Mosaic overlap adapter associates same-class candidates from different intersecting source patches by shared foreground-pixel contact in the patch intersection and merges accepted pairs with Union-Find. SMM is evaluated as the recorded class-wise graph/ILP grouping adapter. Exact grouping rules, score aggregation, parameter values, and implementation-specific limitations for both reference adapters are documented in the Supplementary Material, Section S-VII. The SMM row should therefore be interpreted as the evaluated adapter configuration rather than as a claim about all possible SMM formulations. A separate non-overlapping useful-area protocol is also reported in Section S-VII. The overlapping SMM comparison and its 20-setting sweep use the corrected adapter with within-group all-pairs compatibility constraints. Exhaustive checks of all unordered pairs in every final group found zero incompatible pairs, including pairs without a direct graph edge. The SAM-Mosaic anchor counts are confirmed by the measured association sweep in Supplementary Section S-XX.

Table 5 reports the fixed-candidate consolidation results under the nominal 20% overlap protocol.

DSC consolidation has the highest precision and F1 among the five controlled consolidation-stage configurations. Direct has the highest recall among those five controls but also the largest false-discovery rate; the SMM and SAM-Mosaic reference adapters achieve slightly higher recall (0.9670) but substantially lower precision and F1 than DSC. Relative to the closest controlled comparator, Box NMS + box-IoS, DSC reduces FP from 288 to 186 and changes TP from 1668 to 1662, increasing F1 from 0.8989 to 0.9226 at a small recall cost (0.9504 to 0.9470). Relative to Box NMS, DSC consolidation improves precision by 0.3994 and F1 by 0.2657. The SMM and overlap-adapted SAM-Mosaic rows are reference adapter comparisons under the same candidate protocol and are not used as the primary summary comparison. The corrected SMM result satisfies the reported all-pairs output check; the comparison remains specific to the shared candidate generator and tested parameter settings.

#### 4.2.3. Sensitivity to the Two Consolidation Thresholds

**Figure 5:**
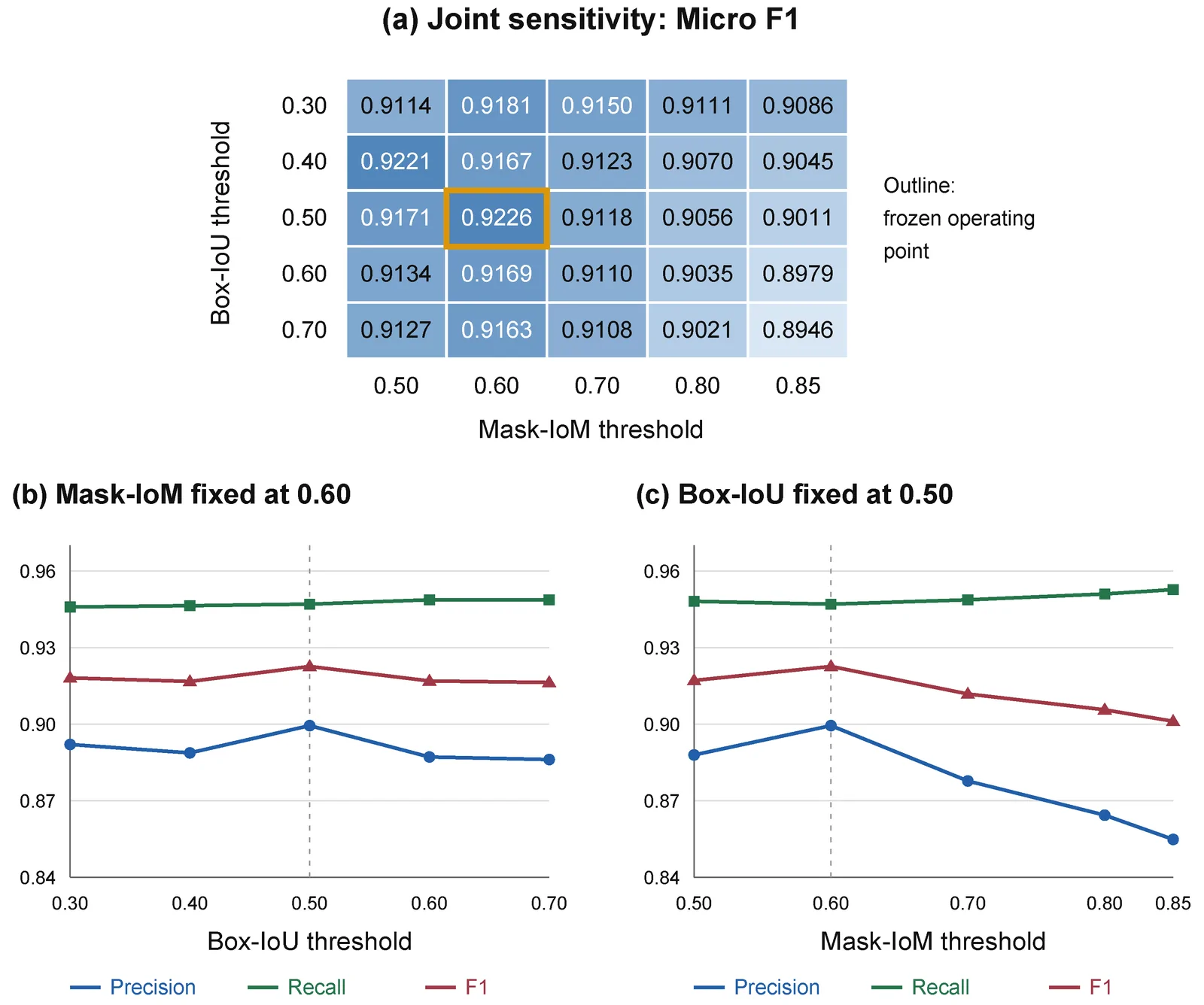
Post-selection DSC threshold sensitivity on fixed candidates, pooled across all classes. (**a**) Micro F1 over the 25 threshold pairs. The outlined cell is the frozen operating point. (**b**,**c**) Precision, recall, and F1 along the two operating-point slices. Lines connect measured settings and do not imply interpolation or confidence intervals.

We examined $\tau_{b}=\tau_{\mathrm{IoU}}\in \left\{0.30,0.40,0.50,0.60,0.70\right\}$ and $\tau_{c}=\tau_{\mathrm{IoM}}\in \left\{0.50,0.60,0.70,0.80,0.85\right\}$ on the ten test drawings after fixing the operating point on validation data. The checkpoint, 20% overlap, confidence cutoff, candidate ordering, and evaluator were held fixed. Figure 5 reports all 25 F1 values and the precision–recall profiles along the two slices through (0.50,0.60). Supplementary Section S-XVII provides the slice counts and grid values. These are post-selection diagnostics, not a second threshold-selection procedure.

At $\tau_{c}=0.60$, varying $\tau_{b}$ from 0.30 to 0.70 gives F1 values of 0.9163–0.9226 and recall of 0.9459–0.9487. At $\tau_{b}=0.50$, increasing $\tau_{c}$ from 0.60 to 0.85 raises TP from 1662 to 1672 but FP from 186 to 284: recall rises from 0.9470 to 0.9527, precision falls from 0.8994 to 0.8548, and F1 falls to 0.9011. A more permissive containment threshold therefore retains a few additional matches at a much larger false-positive cost in this range. Lowering $\tau_{c}$ to 0.50 gives F1 0.9171. Greedy suppression changes which candidates remain available to suppress later candidates, so the retained sets and metrics need not vary monotonically with the threshold.

Across the full grid, F1 ranges from 0.8946 to 0.9226. The tested $\tau_{b}=0.40$–0.60, $\tau_{c}=0.50$–0.70 neighborhood spans 0.9110–0.9226; this supports limited local tolerance, not threshold invariance. The test maximum occurs at the already frozen operating point, but that observation does not establish a universal optimum. These curves quantify the aggregate threshold response across all categories. The following class-specific analysis resolves the responses of three selected categories, while the later error and suppression audit describes residual errors at the fixed (0.50,0.60) operating point.

#### 4.2.4. Category-Specific Threshold Responses

**Figure 6:**
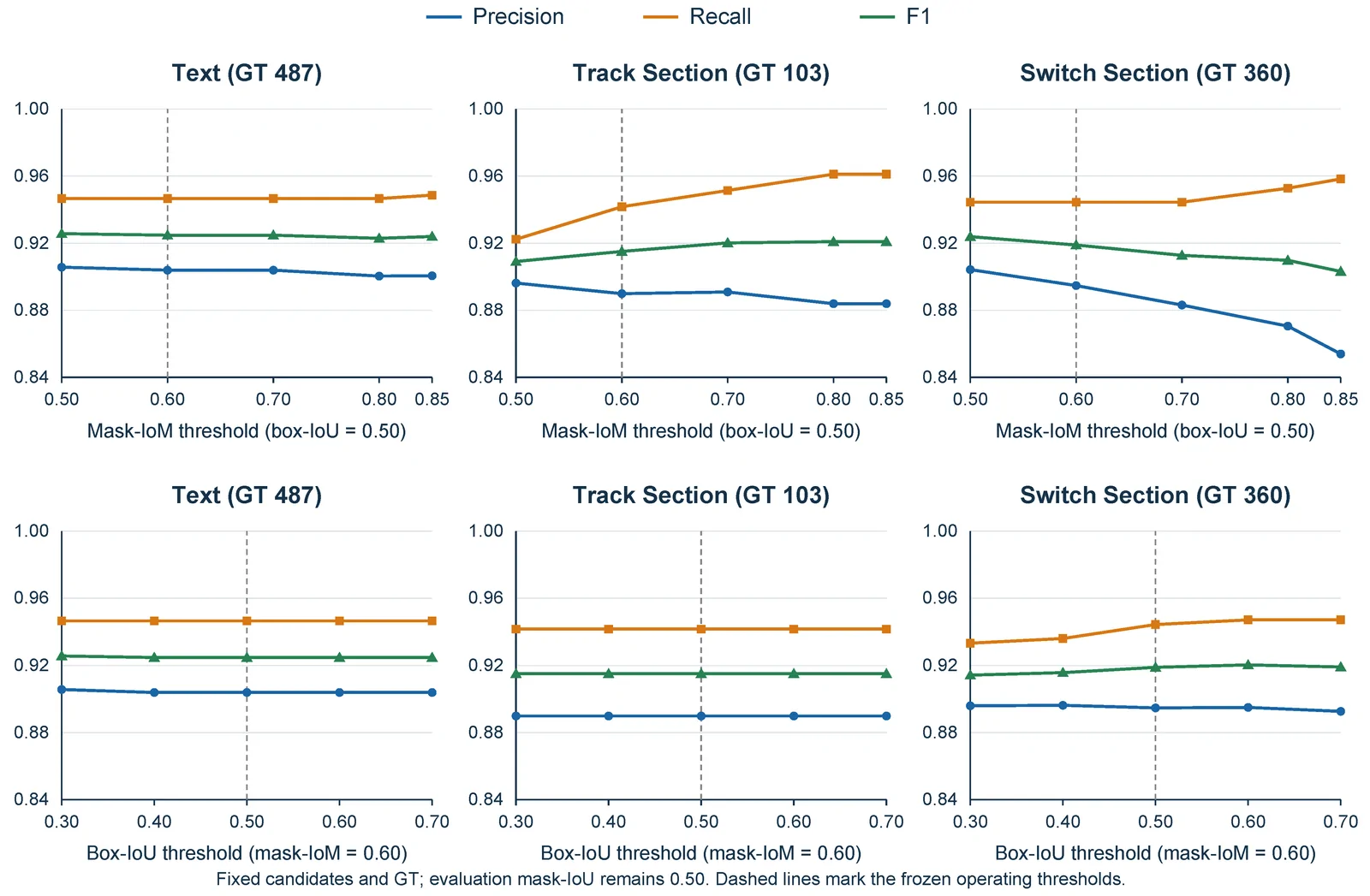
Measured threshold responses for three selected classes. Top: vary mask-IoM at fixed box-IoU 0.50. Bottom: vary box-IoU at fixed mask-IoM 0.60. Curves join the measured points; all panels share the same vertical scale. Vertical dashed lines mark the frozen operating value. The thresholds control consolidation; the evaluation mask-IoU matching threshold remains 0.50.

Figure 6 resolves the two operating-point slices for Text (GT=487), Track Section (GT=103), and Switch Section (GT=360), categories relevant to the suppression audit. These are measured fixed-candidate results. At every threshold, their TP/FP/FN plus the pooled counts of the remaining seven categories (GT=805) reproduce the corresponding whole-drawing slice exactly. The selected point reproduces TP/FP/FN=1662/186/93. Supplementary Section S-XXI provides the counts and metrics.

With box-IoU fixed at 0.50, increasing mask-IoM from 0.50 to 0.85 raises Track Section recall from 0.9223 to 0.9612 and F1 from 0.9091 to 0.9209. Switch Section instead gains recall (0.9444 to 0.9583) while losing precision (0.9043 to 0.8540), reducing F1 from 0.9239 to 0.9031. Text varies less, with F1 between 0.9229 and 0.9257. With mask-IoM fixed at 0.60, Track Section is unchanged across the tested box-IoU thresholds, Text changes by one FP, and Switch Section F1 ranges from 0.9143 to 0.9204. The aggregate optimum therefore does not imply a shared class-wise optimum. These diagnostics support a category-dependent trade-off, but do not establish class-adaptive thresholds: the common validation-selected operating point remains unchanged, and the other seven categories have not been resolved individually.

#### 4.2.5. Sensitivity of the Consolidation Adapters

To broaden the fixed-candidate comparison, we evaluated 20 combinations of the SMM compatibility-weight cutoff γ∈{0.300,0.400,0.476,0.550,0.650} and boundary-distance scale $\tau_{d}\in \left\{3,6,9,12\right\}$ pixels, keeping the other adapter parameters fixed. Supplementary Section S-XVIII reports all counts and metrics. F1 ranges from 0.7553 to 0.7999. The largest observed F1, 0.7999 at $\left(\gamma ,\tau_{d}\right)=\left(0.476,9\right)$ or (0.476,12), is only 0.0004 above the original 0.7995 and remains 0.1227 below DSC. Thus DSC has higher F1 than this SMM-derived adapter throughout the tested grid. This is a descriptive test-set sweep, not a validation-tuned replacement baseline or an exhaustive search. The corrected adapter enforces within-group all-pairs compatibility. All 200 recorded scan runs (20 settings on ten drawings) were recorded as optimal, with zero timeouts and zero incompatible pairs in an exhaustive final-group check. Supplementary Section S-XIX gives the solver-status and timing summaries.

For the SAM-Mosaic overlap adapter, we measured four association strategies—one-way best, bidirectional union best, mutual best, and all contact pairs—at contact thresholds of 1, 5, 10, 25, and 50 shared foreground pixels. The sweep contains 20 settings, each evaluated over all ten drawings with the same frozen candidates, GT, and evaluator as the anchor comparison, without retraining or candidate regeneration. The original bidirectional-union, one-pixel anchor remains F1 0.7625. The largest observed F1 is 0.7656 for one-way best at 10 pixels, compared with 0.7637 for the best bidirectional-union setting, 0.7537 for all contact pairs, and 0.6683 for mutual best. DSC remains higher by approximately 15.70 percentage points than the best tested SAM variant. These are post-selection diagnostics of the overlap adaptation, not tuning of the complete SAM2-based Remote SAMsing pipeline or evidence for untested association rules. Supplementary Section S-XX defines the strategies and reports every count triple.

#### 4.2.6. Application Refinement: OCR and Railway Rules

For completeness, the application refinement applies PaddleOCR-based text recognition and railway-specific rules to the DSC-core output. OCR is applied to text candidates, after which normalized strings are filtered or relabeled as Carrier Frequency/Fouling Point Indicator; section masks are then checked for endpoint connectivity and may be reclassified or removed. If OCR remains empty after the retry, the enabled –keep-ocr-fail branch sets only the OCR text field to an empty string and preserves the candidate’s original class, confidence, box, mask, and all other metadata. The candidate bypasses regex-based recognition, relabeling, and OCR-dependent rejection at this stage, while remaining subject to applicable subsequent spatial rules. Section reclassification and unconnected-section deletion use a 35-pixel endpoint-to-box distance. Mask geometry is not fused or altered; detailed processing rules are given in the Supplementary Material, Section S-XIII. Relative to the three-seed core means, the application refinement changes mean Micro F1 from 0.9226 to 0.9407 (+1.81 pp), Precision from 0.8994 to 0.9323, and Recall from 0.9470 to 0.9493. These are descriptive differences between seed means, separate from the frozen-checkpoint core table. Relabeling can change class-aware matches, so application TP need not be a subset of core TP. Aggregate summaries do not support a paired-effect interval; no application bootstrap interval is reported because its resampling record is incomplete.

Table 6 summarizes the refined-output metrics after DSC-core consolidation, OCR, and railway-specific rules, reported as mean ± sample SD across three training seeds.

#### 4.2.7. Sensitivity to Tile Overlap

The checkpoint, confidence threshold, matching rule, and consolidation parameters are held fixed while overlap changes the observed patches and candidates. These test-set sensitivity rows do not redefine the validation-selected 20% operating point. Table 7 reports the held-out sensitivity values. Fixed-candidate comparison changes only the merging rule; overlap sensitivity reruns candidate generation with different tile origins. Neither comparison is used as a dedicated test-time tile-origin invariance experiment.

#### 4.2.8. Quantitative Residual Errors and Suppression Risks

The frozen DSC core leaves 186 FP and 93 FN. The descriptive error tally in Supplementary Section S-X separates final-output association patterns: 75 FP have no annotated-target association, 68 have insufficient overlap with a same-class target, 40 overlap a different-class target, and three are same-class duplicates. For FN, 75 have an insufficient-overlap same-class prediction, 11 have no associated final prediction, and seven have a wrong-class prediction; no one-to-one matching conflicts are recorded. These are disjoint tally categories within FP and FN, respectively, not independently verified physical causes. An insufficient-overlap prediction and its missed target may contribute to both an FP and an FN, so the two totals must not be interpreted as independent failure events.

Text, switch section, and joint account for 118/186 FP (63.44%) and 61/93 FN (65.59%). The insufficient-overlap categories account for 36.56% of FP and 80.65% of FN, whereas only 1.61% of residual FP are labelled same-class duplicates. The remaining error burden therefore motivates improved candidate masks and class discrimination, rather than simply more aggressive duplicate suppression. Absolute error shares also reflect class prevalence; Table 4 supplies GT counts and class-normalized precision and recall.

The second-stage audit records 1512 suppressed candidates, including 120 potential-risk candidates (7.94%). The count closes exactly with the controlled outputs: box NMS retains 1680+1680=3360 candidates and DSC retains 1662+186=1848, a difference of 1512. A risk candidate reaches the class-aware matching threshold for a GT mask while its keeper does not; another retained candidate may nevertheless match that GT. The new class-wise before/after match tally reports 18 GT instances losing a match after mask-IoM; its total closes with the TP change from 1680 after box NMS to 1662 after DSC under fixed candidate geometry, scores, and matching order. Nine losses are switch sections and five are track sections, together 14/18 (77.78%); they account for 9/20 and 5/6 of those classes’ final FN, respectively. Thus 120 is a candidate-risk count, whereas 18 is the measured GT match-loss count; the latter is part of the final 93 FN, not an additional error total. Track and switch sections contribute 95/120 risk candidates (79.17%); their within-class risk fractions are 37/298 (12.42%) and 58/787 (7.37%), respectively. Text contributes 17/124 (13.71%). Zero observed risk among nine removed Carrier Frequency candidates provides little evidence about general safety, and no risk fraction is defined for classes with no suppressed candidates. Full class-wise counts appear in Supplementary Section S-X.

**Figure 7:**
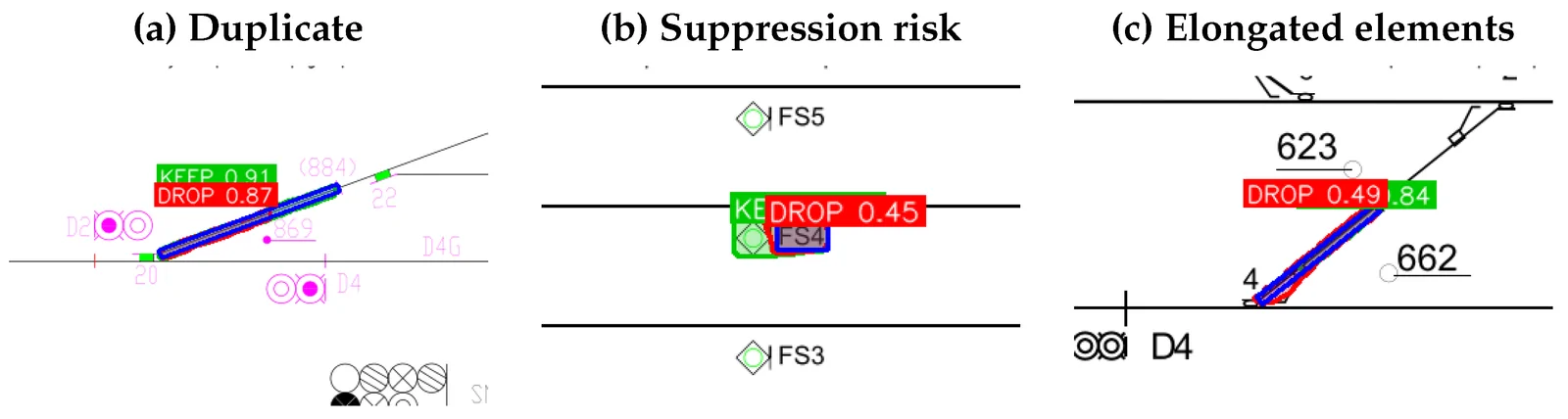
Representative DSC consolidation decisions: (**a**) a matching-status duplicate; (**b**) a potential independent-instance suppression risk; and (**c**) a dense elongated-element case. Green identifies the retained candidate, red the suppressed candidate, and blue the corresponding ground truth where available. Original candidate overlays are retained. The cases illustrate decisions and potential risks, not causal error rates.

These observations explain the precision–recall trade-off without assigning a causal fragment-removal rate. A lower containment threshold can reject duplicate fragments but also remove a valid observation near another same-class element; a higher threshold can preserve such observations while retaining duplicates. Thin track and switch sections provide many opportunities for unequal-extent containment, whereas dense text and compact symbols can be sensitive to small changes in mask support. These geometric interpretations complement the measured threshold responses for Text, Track Section, and Switch Section in Figure 6; the other seven classes are available only as pooled threshold counts. Figure 7 illustrates the corresponding decisions: green denotes the keeper, red the suppressed candidate, and blue the GT where available.

### 4.3. Local-Recognition Ablations

Table 8 places the local predictor in the context of retained results from other instance-segmentation architectures on the same validation-patch split. Principal controls include patch size, overlap, data split, batch-size setting, epoch budget, and early-stopping patience. Exact package revisions and run-level records for the historical cross-architecture runs are unavailable, so this table gives their retained means. RF-DETR-Seg-L is the baseline without Copy–Paste; DSC-Seg is the complete adapted local predictor. Available three-seed RF-DETR summaries are reported separately below, preventing the historical comparison from being confused with the controlled component ablations.

The local adaptations are evaluated with a shared Copy–Paste training configuration. Table 9 reports validation-patch local-recognition performance, with individual and combined components summarized as mean ± sample SD across three training seeds. These results describe local AP changes and do not by themselves establish that each component improves drawing-level F1 after consolidation. Because the same validation split selects checkpoints, these AP values are development-set diagnostics; the held-out drawing-level evaluation is the primary generalization result. RandomShift provides the largest mean Mask AP gain among the reported single additions: 0.6597±0.0017 versus 0.6320±0.0028 for Baseline + CP. The complete representation block reaches 0.6642±0.0014, and adding gated Wasserstein localization gives 0.6782±0.0011. RandomShift is motivated by variation in input-grid alignment, without a claim of complete phase invariance.

The reported SD is the sample standard deviation, $s=\sqrt{\sum_{i=1}^{3}{\left(x_{i}-\bar{x}\right)}^{2}/\left(3-1\right)}$, for seeds 2026, 2027, and 2028, using each run’s best validation-selected EMA checkpoint. Weight Surgery changes mean Mask AP from 0.6377±0.0015 to 0.6390±0.0020 at K=4 without RandomShift, and from 0.6616±0.0037 to 0.6642±0.0014 with RandomShift. These small differences do not establish statistical significance. Adding gated Wasserstein localization to the full representation bundle changes Mask AP from 0.6642±0.0014 to 0.6782±0.0011. Three seeds characterize observed training variability; unavailable paired run-level values prevent a paired-effect interval, and seed dispersion is distinct from drawing-bootstrap uncertainty.

The next table isolates Copy–Paste across three seeds. Subsequent single-run diagnostics examine rare-class AP, elongated-class AP, and compact-target centroid residuals; they complement the repeated-run results without establishing class-specific effects across seeds.

#### 4.3.1. Directed Copy–Paste Across Training Seeds

Directed Copy–Paste increases mean Mask AP from 0.6010±0.0036 to 0.6320±0.0028, a gain of 3.10 percentage points (Table 10). Mean Box AP increases by 2.94 percentage points. The Mask AP difference is larger than either configuration’s observed seed SD, supporting a descriptive overall benefit, without a formal significance claim. The class-specific results below remain single-run diagnostics: they cannot establish that every rare category benefits consistently across seeds.

#### 4.3.2. Directed Copy–Paste: Single-Run Diagnostic

*Evaluation focus:* rare-class Mask AP.

The RF-DETR-Seg-Large baseline is trained with and without directed Copy–Paste. Original source drawings are assigned to splits before augmentation, and pasted instances are introduced only into training crops. The recorded augmentation scale and source restrictions are given in Table 1. Table 11 reports the overall and rare-class Mask AP under the two settings.

The single-run comparison shows an overall Mask AP increase of 3.07 pp and a Carrier Frequency increase of 36.40 pp. The signal-tower decrease (−2.79 pp) indicates a class-specific tradeoff. These are patch-level recognition effects; the class-distribution panels in Figure 2 describe the unaugmented source drawings.

#### 4.3.3. Representation Bundle: Single-Run Diagnostic

*Evaluation focus:* elongated-class (track section, switch section) Mask AP.

Increasing *K* from 2 to 4 provides additional sampling capacity after training; Weight Surgery preserves the pretrained response before perturbation, and RandomShift varies tile alignment. Table 12 reports overall and elongated-class Mask AP between the K = 2 baseline and the bundled representation-level configuration (K = 4 + Surgery + RandomShift).

Track-section Mask AP improves by 2.54 pp and switch-section by 2.19 pp for the complete K=4 + Weight Surgery + RandomShift bundle. This pattern is consistent with the combined design motivation of increasing sampling capacity while varying tile alignment. These class-wise results do not isolate the contributions of *K* expansion, initialization, and RandomShift.

#### 4.3.4. Gated Wasserstein Localization: Single-Run Diagnostic

*Evaluation focus:* compact-class centroid error.

For each matched binary mask, its centroid is the arithmetic mean of its foreground-pixel coordinates, and the residual is the Euclidean distance between prediction and GT centroids in pixels. Matching is class-aware at mask IoU ≥0.5.

Gated Wasserstein localization is evaluated against a *K* = 4 + Surgery + RandomShift baseline while keeping the representation-level setting fixed. Table 13 reports the conditional per-class centroid error (median over each configuration’s matched set at IoU ≥ 0.5) for three selected compact-target categories. Because the auxiliary term can change which instances are matched, the reported residuals are conditional on each configuration’s matched set, and the comparison should be interpreted as the joint effect on matching and localization rather than as a pure localization-only contrast.

Gated Wasserstein localization is associated with a lower per-category median centroid error on the three selected compact categories by 0.19–0.27 px; the final column is only the arithmetic mean of those three medians, not a pooled-instance median. These conditional changes are consistent with the intended localization role, but do not establish subpixel physical accuracy or repeatability across seeds. In the per-category analysis, the complete representation-plus-localization configuration improves Joint Box AP from 0.4791 to 0.5096 and Carrier Frequency Box AP from 0.6176 to 0.6869 relative to Baseline+CP (Supplementary Material, Section S-XIV); Repeater Signal remains a very rare category and is therefore interpreted through the class-wise diagnostic statistics rather than as a standalone claim.

### 4.4. Reliability and Uncertainty

Table 14 separates two sources of uncertainty. For the frozen seed-2026 core, the ten complete drawings are resampled with replacement 10,000 times, with ten drawing IDs per resample. Micro metrics are recomputed from resampled pooled counts and Macro metrics from the resampled per-drawing values. NumPy’s PCG64 generator uses seed 2026; two-sided 95% intervals are the 2.5th and 97.5th percentiles with linear quantile interpolation. The resulting Micro F1 interval is [0.9210, 0.9252]. Neither overlapping patches nor individual instances are independently resampled. These intervals describe drawing-sampling variability within the ten-drawing test set and do not measure uncertainty across railway bureaus or all future drawings. They are intervals for DSC itself, not paired intervals for its performance difference from Box-IoS; the reported 2.36-percentage-point gain is a descriptive difference in pooled test results.

Three-seed summaries instead evaluate each validation-selected EMA checkpoint under the same fixed protocol and report the mean and sample SD; predictions are not pooled across seeds. The primary frozen Macro Precision/FDR (0.8967/0.1033) differ slightly from the core seed means (0.8966/0.1034). Aggregate statistics are available for all three seeds, whereas detailed run artifacts needed for paired seed-level reanalysis are available only for the primary seed-2026 experiment; paired-effect intervals and seed-specific drawing bootstraps are therefore not reported. Core bootstrap intervals are not transferred to the refined output. Per-drawing counts and the evaluation pseudocode remain in the Supplementary Material.

### 4.5. Computational Feasibility

Timing uses batch size 1 with mixed precision disabled. Patch latency is the median of 100 forward passes after ten warm-up passes, with explicit GPU synchronization before and after each timed pass.

The 3.7 s full-application time is the reported median over ten held-out drawings and includes patch generation, recognition, projection, DSC consolidation, OCR, and rule processing. DXF rendering and disk I/O are excluded. The patch-latency rows are labeled by the baseline and full local segmentation configurations in Table 15; the parameter column counts only the local segmentation models. No total parameter count is assigned to the full application pipeline, which also includes PaddleOCR and rule code.

Table 16 reports the fixed-candidate accuracy and repeated timing comparison.

For method *a*, repetition *r*, and drawing *d*, let $t_{a,r,d}$ denote the measured time. We first calculate $m_{a,r}={\mathrm{median}}_{d=1,\ldots ,10}t_{a,r,d}$ and then report ${\mathrm{median}}_{r=1,\ldots ,5}m_{a,r}$. Sample SD (denominator 5−1) and IQR describe the five repetition medians, not drawing-to-drawing variability. Core time is measured directly for each pipeline rather than calculated by adding stage medians. The separate 3.7 s full-application result includes OCR and railway rules and uses a different summary scope.

Box NMS + box-IoS has the lowest recorded time and F1 0.8989. DSC raises F1 by approximately 2.36 percentage points with a 0.6528 s higher core summary. DSC’s merge summary is lower than that of the SAM-Mosaic overlap adapter (0.7127 versus 0.9492 s); their core summaries are much closer (1.8724 versus 1.8968 s). The core sample SDs across repetition medians are 0.040277 and 0.137013 s, respectively. The observed 0.024426 s difference, approximately 1.29% of the SAM-Mosaic core summary, is descriptive and does not establish a statistically significant speed advantage. The corrected SMM adapter has merge and core summaries of 4.8708 and 5.9227 s.

The rented resource-measurement instance uses an AMD EPYC 7K62 48-Core Processor host with 24 allocated CPU cores, platform-advertised system memory of 62G, and a GPU designated by the rental platform as “4090-48G” (48G GPU memory). The allocated core count does not specify the software thread count. Earlier CPU peak-RSS measurements were 2493 MiB for box-IoS, 2692 MiB for DSC, 3216 MiB for the earlier SMM adapter, and 2476 MiB for SAM-Mosaic. These are retained as separate earlier measurements; 3216 MiB is not assigned to the corrected SMM timing runs. CPU RSS is distinct from GPU memory capacity and peak usage. Software thread settings, measured GPU-memory peaks, and RSS sampling details remain unavailable. The comparison is descriptive for these implementations, not complete independently trained systems or all tiling pipelines.

### 4.6. Qualitative Consolidation

**Figure 8:**
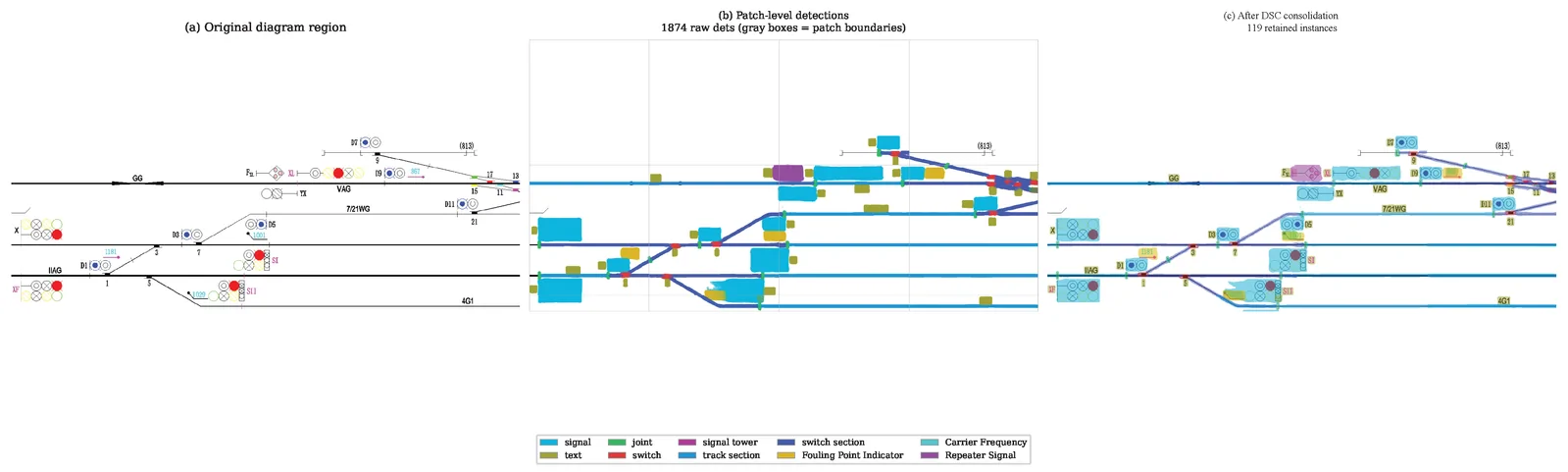
Qualitative example of document-space consolidation: (**a**) the original diagram region; (**b**) projected patch-level predictions before consolidation; and (**c**) the retained DSC-core instances after two-stage consolidation.

Figure 8 shows the original diagram region, projected patch predictions, and consolidated output. DSC consolidation selects existing masks and leaves the retained geometry unchanged. The Supplementary Material, Section S-XIV, additionally visualizes errors after patch predictions are directly projected onto the full drawing, before any drawing-level post-processing. This example motivates document-space consolidation; its quantitative benefit is evaluated by the fixed-candidate comparisons. Figure 4 provides a full-drawing example; additional local comparisons are provided in the Supplementary Material.

## 5. Discussion

DSC consolidation addresses unequal-extent observations by comparing overlap against the smaller mask’s support. Under the selected 20% protocol, DSC has higher precision and lower recall than the SMM and SAM-Mosaic adapters at their anchor settings. The corrected SMM comparison includes 20 settings with all-pairs compatibility enforced and zero incompatible pairs in the final groups. These results apply to the evaluated implementation and parameter grid; they do not establish superiority across other candidate generators or untested SMM settings. The 20-setting SAM-Mosaic overlap sweep also leaves DSC with higher F1 than every tested variant, while showing that the association rule materially changes the precision–recall balance. Both conclusions remain specific to the shared candidates and tested adapters. The ranking reverses for the non-overlapping useful-area protocol: SAM-Mosaic reaches F1 0.8877 and DSC consolidation 0.8030, compared with 0.7625 and 0.9226 under overlap. A mechanism-level interpretation is that non-overlapping useful areas provide no shared mask support between adjacent regions, limiting IoM’s ability to associate cross-region fragments, whereas the useful-area adapter constructs class-wise label rasters with confidence-prioritized pixel ownership and uses bidirectional boundary best-match association without requiring mutual agreement. This is an interpretation of the protocol design, not an isolated causal claim, because useful-area cropping, context padding, confidence filtering, and adapter behavior change simultaneously.

The fixed-candidate controls separate Direct, box-NMS-only, mask-IoM-only, box-IoS, and complete DSC consolidation. They show a reduction in false positives under the selected candidate protocol, while also showing the associated small recall loss. Box NMS + box-IoS and DSC consolidation (box NMS + mask-IoM) use the same box-NMS threshold (0.5) and containment threshold (0.6), enabling a matched-parameter comparison of box and mask support. This comparison isolates the choice of box or mask support at the stated numerical thresholds. Box-IoS was not independently tuned across thresholds, so the observed gain is not a comparison between separately optimized operators. The controls evaluate the chosen order against its individual stages; they do not test the reversed order or establish that this sequence is optimal. In particular, a candidate removed by box NMS is not restored if its suppressor is subsequently removed by mask-IoM. The quantitative association and suppression-risk tallies do not establish causal fragment-removal rates: high containment can arise from duplicates, truncation, or independent instances. Confidence-first selection can retain incomplete geometry, and no consolidation can recover geometry absent from all candidates.

The four unseen-source drawings have similar aggregate performance to the six seen-source drawings, but cover only one additional bureau and two maintenance sections. Rare categories, particularly Carrier Frequency and Signal tower, have few test instances. The three directed Copy–Paste target categories were selected from pre-augmentation class-frequency statistics over all 81 source drawings, so this dataset-design decision uses held-out class-count metadata even though no validation/test image or instance is used as augmentation material and no test metric is used for tuning. A stricter future protocol should determine augmentation targets from training-only class frequencies. Broader source-held-out and multi-annotator evaluation is also needed; the private dataset and unavailable production code constrain external replication. Patch AP is specified as validation-patch evaluation with explicit tiling, evaluator slice, and model-side export-cap settings. The held-out drawing-level results remain the primary evidence for whole-drawing performance.

OCR and railway rules refine the inventory after consolidation without providing validated topology reconstruction or signalling-safety assurance. The OCR patterns and regex-generated whitelist, FPI geometry test, endpoint handling for branched masks, and endpoint-to-box connectivity definition are given in the Supplementary Material, Section S-XIII. The 35-pixel connectivity distance is raster-scale dependent, and combined refined-output metrics do not isolate individual rules. These boundaries keep the demonstrated contribution at the level of a document-space instance inventory for engineering review.

### 5.1. Dataset Access and the Limits of Generalization

RSDD is a restricted real-project dataset, so this study is a case study rather than an externally reproducible benchmark. Neither an authorized anonymized subset nor a validated synthetic counterpart is available. The reported protocols, pseudocode, and aggregate results make the analysis more transparent but cannot substitute for access to the images and annotations.

Ten test drawings are too few to characterize the full diversity of drawing conventions, equipment inventories, raster scales, and bureau-specific practices. The 1755 instances are clustered within these drawings and must not be treated as 1755 independent source-level replications. Carrier Frequency and signal tower each have only ten test instances; one additional missed instance changes recall by ten percentage points. The four unseen-source drawings cover only one additional bureau. Similar seen- and unseen-source F1 values are therefore preliminary evidence within this sample, not proof of bureau-invariant performance. Drawing bootstrap intervals and training-seed SD quantify different conditional variations and do not resolve these coverage limitations.

A future multisource evaluation should hold out entire railway bureaus, group drawings from the same station or project to avoid related-document leakage, and reserve a separate validation pool for all model and threshold choices. It should report results by bureau, raster scale, and category, extend rare-category coverage, and include independent annotation review. Augmentation targets should be selected using training-only frequencies. Such an evaluation would test a frozen DSC operator on unseen sources before any local recalibration; results after recalibration should be reported separately. This is a proposed verification protocol, contingent on data-access authorization, rather than an additional experiment completed here.

### 5.2. Transfer to Other Engineering Diagrams

The DSC operator uses class labels, confidence scores, boxes, and masks in a common image coordinate system. These inputs are also available for electrical schematics and piping and instrumentation diagrams, so the projection and two-stage selection procedure can be reused in principle. The local predictor must learn the target domain’s symbols and annotation conventions, and thresholds must be calibrated on its validation drawings. Railroad-specific OCR patterns and connectivity rules would need replacement; the 35-pixel connectivity threshold is neither scale-invariant nor portable.

Transfer is most plausible when overlapping tiles provide competing, substantially overlapping observations of one physical or graphical instance. Wire crossings, nested symbols, and adjacent same-class components can instead produce valid containment and require explicit evaluation of false suppression. The definition of an instance is also consequential: a pipe or wire may be annotated as a complete network, an edge between junctions, or a local segment. Disjoint fragments cannot be recovered by mask containment, and topology reconstruction requires additional association logic. A target-domain evaluation should therefore compare fixed candidates, vary overlap and both thresholds, and measure category-wise errors and connectivity separately. No cross-domain performance is claimed from the railway experiments.

## 6. Conclusions

DSC reconciles unequal-extent observations from overlapping tiles through coordinate projection and ordered box-NMS/mask-IoM selection. On ten held-out railway drawings, the validation-selected configuration achieves precision 0.8994, recall 0.9470, and F1 0.9226. With identical candidates and box-NMS/containment thresholds of 0.50/0.60, DSC yields 102 fewer FP and increases F1 by 2.36 percentage points relative to box-IoS filtering. The experiments distinguish local recognition, consolidation, and subsequent application refinement. Three-seed local ablations, SMM and SAM-Mosaic adapter sweeps, three-class threshold responses, and quantitative error and resource analyses characterize the observed gains and their costs.

The evidence supports a document-space instance inventory under the tested overlapping-tile protocol. It does not establish general superiority under non-overlapping tiling, freedom from false suppression, or transfer across engineering domains. Restricted data access, the small source-level test set, and untested candidate generators remain limitations. Threshold responses are resolved individually for three selected categories, with the other seven reported only in aggregate. Independent multisource testing and domain-specific topology evaluation are required before broader workflow-level claims can be made.

## Figures and Tables

**Table 1:** Recorded training-patch and augmentation scale.

Item	Recorded Scale and Provenance
Original training patches	4813 images, all 768×768 pixels; generated with stride 384 (50% overlap) from 57 training drawings
Final negative patches	1874 images with no retained annotation in the final export, after Copy–Paste; included in the 4813 training images
Copy–Paste	Offline augmentation of 570 existing blank training patches, modified in place and replacing their originals; 780 added instances (380 signal towers, 300 Repeater Signals, 100 Carrier Frequency instances)
Target selection	Three least frequent categories in the pre-augmentation whole-dataset class-frequency statistics (all 81 source drawings): signal tower, Repeater Signal, and Carrier Frequency
Final training export	4813 images: 2939 with retained annotations and 1874 negative patches; Copy–Paste does not increase the number of images
Augmentation source	Pasted instances and background images were drawn only from the training split; no validation or test source was used

**Table 2:** Key operating and training parameters. Values used for held-out evaluation are determined from the training/validation workflow before final test reporting.

Parameter	Value	Basis/Role
Patch size	768×768	Fixed local-predictor input size
Validation-patch stride	384 (50% overlap)	Patch-level AP evaluation and local-recognition ablations
Drawing-level nominal overlap	20% (stride 614; realized regular-grid overlap ≈20.05%)	Selected on the 14 validation drawings by drawing-level Micro F1; ties use higher Recall then lower FDR
Drawing-level confidence threshold	0.20	Fixed candidate-export cutoff in the locked protocol
Box-NMS IoU threshold $\tau_{\mathrm{IoU}}$	0.5	Fixed first-stage DSC operating value
Box-IoS containment threshold	0.6	Fixed comparator value in the controlled box-containment row
Mask-IoM threshold $\tau_{\mathrm{IoM}}$	0.6	Validation-selected recall–FDR operating point
Gated Wasserstein scale *C*	0.1	Compact-target localization setting
Gated Wasserstein aspect-ratio cutoff	7	GT-box morphology gate
Gated Wasserstein normalized-area cutoff	0.04	GT-box morphology gate
Weight-Surgery perturbation $\sigma_{s}/\sigma_{a}$	0.02/0.01	Symmetry breaking after equivalent K=2→4 initialization
RandomShift range	±12 px	Covers every residue class of the 12-pixel input sampling lattice
Repeated-training seeds	2026, 2027, 2028	Mean ± sample SD for repeated-run summaries

**Table 3:** Frozen-checkpoint DSC-core output on ten held-out drawings (1755 GT instances; 768-pixel tiles, stride 614). Micro metrics pool counts; Macro metrics average per-drawing values. This table precedes OCR and railway-specific rule processing.

Pipeline	Avg.	*P*	*R*	FDR	F1
DSC core	Micro	0.8994	0.9470	0.1006	0.9226
DSC core	Macro	0.8967	0.9527	0.1033	0.9238

**Table 4:** DSC-core results by category under the locked 20% protocol. Per-drawing counts are retained in the Supplementary Material, Section S-XII. The Macro row is the unweighted mean over the ten categories and is distinct from the drawing-level Macro row in Table 3.

Class	GT	TP	FP	FN	Precision	Recall	FDR	F1
Signal	227	216	25	11	0.8963	0.9515	0.1037	0.9231
Text	487	461	49	26	0.9039	0.9466	0.0961	0.9248
Joint	294	279	29	15	0.9058	0.9490	0.0942	0.9269
Switch	140	133	15	7	0.8986	0.9500	0.1014	0.9236
Signal tower	10	10	1	0	0.9091	1.0000	0.0909	0.9524
Track section	103	97	12	6	0.8899	0.9417	0.1101	0.9151
Switch section	360	340	40	20	0.8947	0.9444	0.1053	0.9189
Fouling Point Indicator	106	101	11	5	0.9018	0.9528	0.0982	0.9266
Carrier Frequency	10	8	2	2	0.8000	0.8000	0.2000	0.8000
Repeater Signal	18	17	2	1	0.8947	0.9444	0.1053	0.9189
Micro (per-drawing total)	1755	1662	186	93	0.8994	0.9470	0.1006	0.9226
Macro (by class)	–	–	–	–	0.8895	0.9381	0.1105	0.9130

**Table 5:** Fixed-candidate consolidation under nominal 20% overlap. All metrics are Micro. The five consolidation-stage controls and the complete-operator rows use the same candidate export, confidence cutoff, class labels, initial candidate ordering, and class-aware mask-IoU evaluator; each adapter applies its own output-score aggregation and selection rule. The overlap adapter is distinguished from the separate non-overlapping useful-area protocol. SMM and SAM-Mosaic counts match their measured sweep anchors; the SAM-Mosaic anchor uses bidirectional union best with one-pixel minimum contact.

Configuration	Precision	Recall	FDR	F1	TP	FP	FN
Direct	0.3452	0.9630	0.6548	0.5082	1690	3206	65
Box NMS	0.5000	0.9573	0.5000	0.6569	1680	1680	75
Mask-IoM only	0.8214	0.9538	0.1786	0.8827	1674	364	81
Box NMS + box-IoS	0.8528	0.9504	0.1472	0.8989	1668	288	87
DSC	0.8994	0.9470	0.1006	0.9226	1662	186	93
SMM (corrected)	0.6815	0.9670	0.3185	0.7995	1697	793	58
SAM-Mosaic merge adapter (overlap)	0.6295	0.9670	0.3705	0.7625	1697	999	58

**Table 6:** Refined output after DSC core, OCR, and railway-specific rules under the selected 20% protocol. Values are mean ± sample SD across three training seeds (2026–2028). For each seed, Micro metrics pool the ten drawings and Macro metrics average their per-drawing values; the resulting seed metrics are then averaged.

Aggregation	Precision	Recall	FDR	F1
Macro	0.9295±0.0032	0.9549±0.0028	0.0705±0.0032	0.9420±0.0022
Micro	0.9323±0.0028	0.9493±0.0030	0.0677±0.0028	0.9407±0.0020

**Table 7:** DSC-core sensitivity to nominal tile overlap; all metrics are Micro. Terminal boundary alignment can introduce residual edge overlap in the nominal 0% setting. No OCR/rule refinement or retraining is applied.

Overlap	Precision	Recall	FDR	F1
0%	0.8910	0.9459	0.1090	0.9176
20%	**0.8994**	0.9470	**0.1006**	**0.9226**
25%	0.8875	0.9436	0.1125	0.9147
50%	0.8866	**0.9493**	0.1134	0.9169

**Table 8:** Cross-architecture local-recognition comparison on the common validation-patch split. All rows report retained mean metrics from repeated runs under the same principal training controls (patch size, overlap, split, batch-size setting, epoch budget, and early-stopping patience). Exact software revisions and run-level records for the historical architecture comparison were not retained; available RF-DETR three-seed summaries are reported separately in the component ablations. Values are Box AP, Box AP_50_, Box AP_75_, Mask AP, Mask AP_50_, and Mask AP_75_, respectively. Missing entries were unavailable in the retained comparison record. Bold values mark the largest retained value in each column.

Model	Box AP	Box AP_50_	Box AP_75_	Mask AP	Mask AP_50_	Mask AP_75_
YOLO11-seg-L [26]	**0.7228**	0.9195	**0.7840**	0.4751	0.7808	0.5176
YOLO26-seg-L [27]	0.6988	0.9122	0.7766	0.4445	0.7508	0.4740
Mask2Former [28]	– ^†^	–	–	0.6173	0.8449	0.6432
RF-DETR-Seg-L	0.6336	0.8668	0.6932	0.6010	0.8612	0.6505
DSC-Seg	0.7045	**0.9259**	0.7809	**0.6782**	**0.9281**	–

^†^ The Mask2Former Box AP/AP_50_/AP_75_ values and the DSC-Seg Mask AP_75_ value were not retained in the historical comparison record; the corresponding entries are therefore left as dashes.

**Table 9:** Individual and combined local-recognition ablations with repeated-run statistics. Values are mean ± sample standard deviation across seeds 2026, 2027, and 2028; all configurations use the same data split and preprocessing, with directed Copy–Paste enabled in every row.

Configuration	Params	Box AP	Box AP_50_	Box AP_75_	Mask AP	Mask AP_50_
Baseline + CP	35.59M	0.6630 ± 0.0008	0.8919 ± 0.0021	0.7342 ± 0.0066	0.6320 ± 0.0028	0.8828 ± 0.0018
+ RandomShift only	35.59M	0.6882 ± 0.0018	0.9101 ± 0.0054	0.7633 ± 0.0060	0.6597 ± 0.0017	0.9120 ± 0.0055
+ K = 4 default init	35.71M	0.6563 ± 0.0012	0.8873 ± 0.0031	0.7294 ± 0.0027	0.6377 ± 0.0015	0.8921 ± 0.0018
+ K = 4 + RandomShift	35.71M	0.6894 ± 0.0016	0.9153 ± 0.0048	0.7653 ± 0.0029	0.6616 ± 0.0037	0.9086 ± 0.0021
+ K = 4 + Weight Surgery	35.71M	0.6697 ± 0.0006	0.8939 ± 0.0018	0.7442 ± 0.0035	0.6390 ± 0.0020	0.8948 ± 0.0021
+ K = 4 + Weight Surgery + RandomShift	35.71M	0.6934 ± 0.0010	0.9128 ± 0.0026	0.7701 ± 0.0036	0.6642 ± 0.0014	0.9153 ± 0.0039
+ Gated Wasserstein only	35.59M	0.6679 ± 0.0015	0.8892 ± 0.0020	0.7407 ± 0.0011	0.6350 ± 0.0030	0.8899 ± 0.0027
+ K = 4 + Weight Surgery + RandomShift + Gated Wasserstein (Ours)	35.71M	**0.7045 ± 0.0021**	**0.9259 ± 0.0014**	**0.7809 ± 0.0030**	**0.6782 ± 0.0011**	**0.9281 ± 0.0020**

**Table 10:** Three-seed Copy–Paste ablation on the common validation-patch split. Values are mean ± sample SD across seeds 2026–2028. The two configurations differ in directed Copy–Paste; all other baseline settings are shared. Mask AP_75_ with CP was not available in the retained summary.

Configuration	Box AP	Box AP_50_	Box AP_75_	Mask AP	Mask AP_50_	Mask AP_75_
Baseline without CP	0.6336±0.0012	0.8668±0.0022	0.6932±0.0018	0.6010±0.0036	0.8612±0.0025	0.6505±0.0020
Baseline with CP	0.6630±0.0008	0.8919±0.0021	0.7342±0.0066	0.6320±0.0028	0.8828±0.0018	–

**Table 11:** Single-run effect of directed Copy–Paste on overall and rare-class patch-level Mask AP. These descriptive results are distinct from the three-seed main ablation.

Config.	Mask AP	Carrier F.	Signal Tower	Repeater Sig.
Baseline w/o CP	0.6010	0.2536	0.8028	0.7298
Baseline w/ CP	0.6317	0.6176	0.7749	0.7839
Δ (pp)	+3.07	+36.40	−2.79	+5.41

**Table 12:** Single-run effect of the representation-level bundle (*K* expansion + Weight Surgery + tokenization-period-calibrated RandomShift) on elongated-class Mask AP. Both rows include Copy–Paste.

Config.	Mask AP	Track Sec.	Switch Sec.
Baseline + CP (*K* = 2)	0.6317	0.6610	0.5832
+Repr. comp. (*K* = 4 + Surgery + RS)	0.6617	0.6864	0.6051
Δ (pp)	+3.00	+2.54	+2.19

**Table 13:** Single-run gated Wasserstein localization effect on compact-class centroid error (median, in pixels; lower is better). Residuals are conditional on each configuration’s matched set, since enabling the auxiliary term can change which instances are matched at IoU ≥ 0.5.

Config.	Joint	Carrier F.	Repeater Sig.	Mean of Three Medians
+Repr. comp.	1.46	1.61	1.39	1.49
+Repr. comp. + Gated Wasserstein	**1.27**	**1.34**	**1.18**	**1.26**
Δ (px)	−0.19	−0.27	−0.21	−0.23

**Table 14:** DSC-core uncertainty under the selected 20% protocol. Point estimates and drawing-bootstrap intervals refer to the frozen seed-2026 checkpoint. The last column contains previously retained means and sample SDs across seeds 2026–2028; it measures a different source of variation. Detailed run artifacts required for paired seed-level reanalysis are available only for the primary seed-2026 experiment.

Metric	Frozen Point Estimate	Drawing-Bootstrap 95% CI	Three-Seed Mean ± SD
Micro Precision	0.8994	[0.8944, 0.9030]	0.8994±0.0026
Micro Recall	0.9470	[0.9405, 0.9571]	0.9470±0.0030
Micro FDR	0.1006	[0.0970, 0.1056]	0.1006±0.0026
Micro F1	0.9226	[0.9210, 0.9252]	0.9226±0.0020
Macro Precision	0.8967	[0.8930, 0.9007]	0.8966±0.0028
Macro Recall	0.9527	[0.9451, 0.9594]	0.9526±0.0026
Macro FDR	0.1033	[0.0993, 0.1070]	0.1034±0.0028
Macro F1	0.9238	[0.9216, 0.9262]	0.9239±0.0026

**Table 15:** Computational efficiency on an NVIDIA RTX 4090 platform. Patch latency is the median over 100 iterations at 768×768; the full application-pipeline time is the median over the 10 held-out diagrams.

Stage/Setting	Segmentation Params	Patch Latency	Full-Diagram Time
Baseline + CP	35.59M	35.0 ms	—
DSC-Seg local predictor	35.71M	35.0 ms	—
Full application pipeline	—	—	3.7 s

**Table 16:** Accuracy and repeated timing under the fixed-candidate comparison. Each repetition processes ten drawings; each time is the median across five repetition-level per-drawing medians (seconds per drawing), not the pooled median of 50 drawing runs. Merge and core times are measured separately. SD, IQR, and core repetition values are in Supplementary Section S-XIX. Accuracy is before OCR and railway rules.

Adapter	*P*	*R*	F1	Merge Time (s/Drawing)	Core Time (s/Drawing)
Box NMS + box-IoS	0.8528	0.9504	0.8989	0.0106	1.2196
DSC	0.8994	0.9470	0.9226	0.7127	1.8724
SMM (corrected)	0.6815	0.9670	0.7995	4.8708	5.9227
SAM-Mosaic (overlap)	0.6295	0.9670	0.7625	0.9492	1.8968

## Data Availability

The source drawings, corrected annotations, production checkpoints, and production inference code are unavailable for public distribution under proprietary railway-project restrictions. No authorized anonymized subset or validated synthetic dataset is available. The manuscript and Supplementary Material contain the reported protocols, pseudocode, threshold analyses, and anonymized aggregate and per-drawing results. These materials support procedural transparency but do not enable independent reproduction of the private-data numerical results.

## Supplementary Materials

The following supporting information can be downloaded at: https://www.mdpi.com/article/10.3390/s26206413/s1, The Supplementary Material provides full-drawing visualizations, annotation and evaluation protocols, the Weight Surgery numerical check, baseline-adapter definitions, SMM and SAM-Mosaic sweeps, joint and class-specific threshold results, residual-error and per-drawing counts, repeated timing and solver-status records, local-recognition diagnostics, OCR and railway-rule specifications, and algorithm pseudocode.
