# Supplementary material for: DSC: Containment-Aware Instance Consolidation for Ultra-High-Resolution Railway Signal-Layout Image Analysis
Source: Sensors (Basel). 2026 Oct 10;26(20):6413. doi: 10.3390/s26206413 (PMC13709312; doi:10.3390/s26206413)
Supplement: Supplementary file 1 [file sensors-26-06413-s001.zip › Definitions/logo-mdpi-eps-converted-to.pdf]

MDPI
