# Supplementary material for: DSC: Containment-Aware Instance Consolidation for Ultra-High-Resolution Railway Signal-Layout Image Analysis
Source: Sensors (Basel). 2026 Oct 10;26(20):6413. doi: 10.3390/s26206413 (PMC13709312; doi:10.3390/s26206413)
Supplement: Supplementary file 1 [file sensors-26-06413-s001.zip › geometric_validity.pdf]

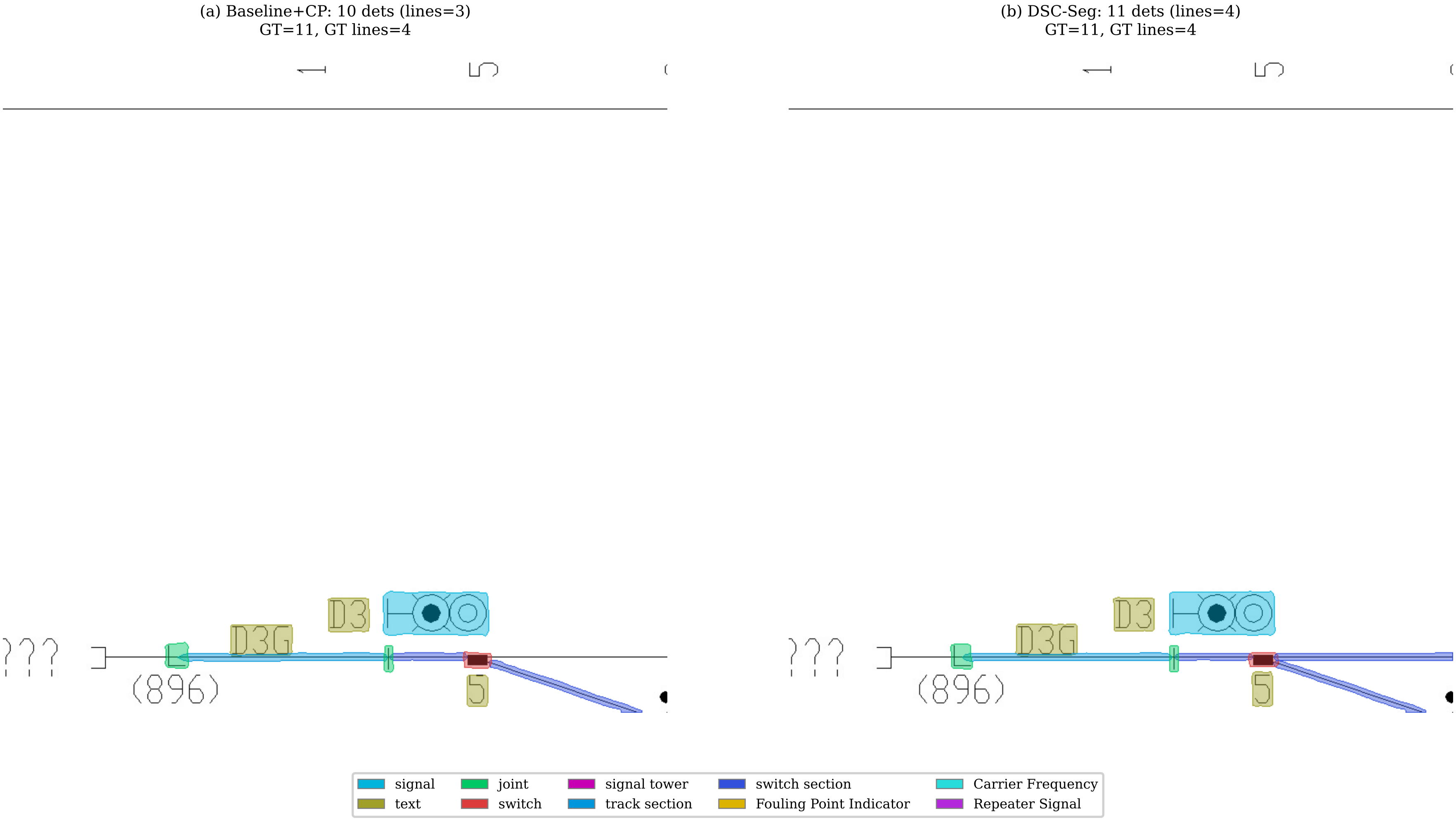
(a) Baseline+CP: 10 dets (lines=3)
GT=11, GT lines=4
D3
D3G
(896)
5
(b) DSC-Seg: 11 dets (lines=4)
GT=11, GT lines=4
D3
D3G
(896)
5
signal
text
joint
switch
signal tower
track section
switch section
Fouling Point Indicator
Carrier Frequency
Repeater Signal
