# Supplementary material for: DSC: Containment-Aware Instance Consolidation for Ultra-High-Resolution Railway Signal-Layout Image Analysis
Source: Sensors (Basel). 2026 Oct 10;26(20):6413. doi: 10.3390/s26206413 (PMC13709312; doi:10.3390/s26206413)
Supplement: Supplementary file 1 [file sensors-26-06413-s001.zip › sensors-4590778-supplementary edited.pdf]

Supplementary Materials: DSC: Containment-Aware Instance Consolidation for Ultra-High-Resolution Railway Signal-Layout Image Analysis

Wenzhen Kuang [1,2], Xixuan Tang [1,2,*], Xiao Fu [2,3] and Wei Xi [2,3]

* Correspondence: Xixuan Tang; txxuan_x@163.com

S-I. Full-Diagram Inference Visualization

Fig. S1 provides an additional full-diagram DSC-core visualization. It uses the same evaluation protocol as the main-paper core result; the application refinement is not applied.

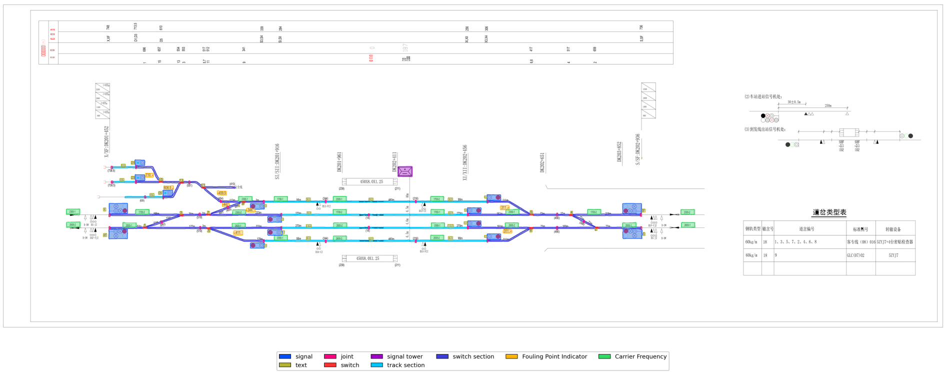

Figure S1. Additional full-diagram visualization of the DSC core (not shown in the main paper), produced with the same checkpoint and two-stage containment-aware consolidation protocol (box NMS followed by mask-IoM suppression) as the core quantitative evaluation. Color coding follows the class legend used in the main manuscript.

S-II. Dataset Provenance and Annotation Audit

The main-paper dataset section specifies the drawing split, annotation construction, training-time clipping rules, and final Copy–Paste export. The pre-augmentation class-frequency statistics across all 81 complete source drawings are long-tailed; signal tower, Repeater Signal, and Carrier Frequency are the three least frequent categories in these dataset-level statistics and are therefore selected as the directed Copy–Paste targets. This target selection uses dataset-level class-count metadata, whereas the augmentation operation itself uses only training-split instances and background patches. The split is by complete source drawing; no source drawing crosses training, validation, and test. Training/validation covers three bureaus and five maintenance sections. The ten held-out drawings comprise six represented-source drawings and four drawings from one additional bureau and two additional maintenance sections. Anonymous per-drawing counts and the source-stratified diagnostic are retained in Section S-XII.

All ten test drawings were independently reviewed by a railway-signalling-trained annotator. Five instance-level errors across three drawings were corrected, and all reported results use that corrected annotation version. Only corrected files remain; the audit is a correction log rather than a recoverable before/after release. Source drawings, annotations, and production code are proprietary (Section S-XVI).

S-III. Weight Surgery: Numerical Verification

The equivalence construction and proof are given in the main-paper representation-level method. This section records the retained numerical implementation check.

Numerical verification. The numerical check in `weight_surgery_diagnosis_small_seed2026.json` compares the original $K = 2$ and expanded $K = 4$ modules on a fixed small input in evaluation mode with identical tensors, shared non-expanded parameters, zero perturbation, and deterministic execution. The stored float32 check reports a maximum absolute output difference of approximately 4.77×10^{-7} (below the configured 10^{-5} tolerance). This is an implementation check up to single-precision round-off, not a float64 10^{-12} guarantee; the algebraic equivalence proved in the main paper is exact before numerical perturbation.

S-IV. Mask-IoM Suppression and Consolidation Pseudocode

The two-stage containment-aware consolidation operator applies class-wise box NMS ($\tau_b = 0.5$) followed by greedy mask-IoM suppression ($\tau_c = 0.6$). Detector-emitted binary masks are resized by nearest-neighbour interpolation if needed, projected, clipped, and discarded if empty; no second logit threshold is applied. Priority is descending confidence and ascending original index (tile traversal followed by detector order), preserved after NMS. Masks, rather than boxes, define $\mathrm{IoM}(m_i, m_j) = |m_i \cap m_j| / \min(|m_i|, |m_j|)$.

Algorithm S1 specifies full inference, and Algorithm S2 specifies the second stage. Tile provenance, adjacency, boundary contact, mask area, and completeness do not determine priority; retained masks remain unchanged.

S-V. Geometric Residual Definitions and Implementation

This section defines the geometric residuals used in the numerical localization tables. These statistics are computed on matched instance pairs (mask IoU ≥ 0.5, class-aware greedy matching). The unprocessed full-drawing overlay in Fig. S3 is a separate qualitative illustration of directly projected patch predictions.

Centroid. The centroid of a binary mask $\hat{\mathbf{m}} \in \{0,1\}^{H \times W}$ is computed as:

$$(c_x, c_y) = \left(\frac{\sum_{i,j} j\, \hat{m}_{ij}}{\sum_{i,j} \hat{m}_{ij}}, \frac{\sum_{i,j} i\, \hat{m}_{ij}}{\sum_{i,j} \hat{m}_{ij}} \right), \tag{S1}$$

where (i, j) are row and column indices. The centroid error for a matched pair is $e_c = \|\mathbf{c}_{\mathrm{pred}} - \mathbf{c}_{\mathrm{gt}}\|_2$. Let $\Delta c_x = c_x^{\mathrm{pred}} - c_x^{\mathrm{gt}}$ and $\Delta c_y = c_y^{\mathrm{pred}} - c_y^{\mathrm{gt}}$. The localization table reports the sample means $\bar{\Delta c}_x$ and $\bar{\Delta c}_y$ and the corresponding sample standard deviations s_{c_x} and s_{c_y}.

Centroid residuals are computed on matched masks. Patch-observation counts can include several observations of one full-drawing instance and are not counts of independent drawing-level GT instances.

S-VI. Training and Runtime Configuration

Optimizer: AdamW with weight decay 1×10^{-4}.
Learning rates: Encoder 5×10^{-5}; decoder and mask head 1×10^{-4} ($2\times$ encoder rate).
Gated Wasserstein localization term: aspect ratio is $\max(w/h, h/w)$ for the normalized patch-level GT box; normalized area is the GT box area wh in normalized coordinates (equivalently, GT box area divided by $H_{\mathrm{patch}} W_{\mathrm{patch}}$). The same gate is applied in Hungarian matching and the regression loss, using aspect ratio < 7 and area < 0.04, and it uses

GT box geometry rather than mask area. Samples outside the gate receive zero auxiliary loss and zero auxiliary matching cost. The implementation uses the squared-Wasserstein similarity $S_{\mathrm{sqW}} = \exp(-W_2^2/C)$ without a square root; $C = 0.1$, the regression coefficient is 1.0, and the matching-cost coefficient is 2.0. This auxiliary term is motivated by NWD but is defined and named here as gated Wasserstein localization.
Scheduler: Step decay at epoch 55 by factor 0.1.
Gradient clipping: Max norm 0.1.
Early stopping: Monitored on validation-set Mask AP with patience 20 epochs.
Pretrained checkpoint: Recorded as official RF-DETR-Seg-Large weights; the study configuration uses a windowed DINOv2-style backbone with patch_size = stride = 12. The exact release identifier and checkpoint hash are unavailable in the configuration record.
Naming: RF-DETR-Seg-L is the table abbreviation for RF-DETR-Seg-Large.

Software environment: PyTorch 2.4, CUDA 12.1, Python 3.10, on Ubuntu 22.04. The model configuration, checkpoint identifier, inference thresholds, consolidation parameters, and evaluation definitions are specified for procedural reproducibility.
Parameter counts: 35.59 M parameters reported for the study-configured RF-DETR-Seg-Large-based baseline; 35.71 M parameters after the K=2 → 4 deformable-attention expansion in DSC (the increase is dominated by the duplicated sampling-offset and attention-weight projections in the decoder cross-attention layers).
Backbone tokenization: The reported study configuration uses a 12-pixel patch embedding stride. Stride $614 \equiv 2 \pmod{12}$ visits multiple input-grid phases; RandomShift over $[-12, 12]$ covers every residue class.

Random seeds: 2026, 2027, and 2028 for the repeated training configurations. The primary frozen core and fixed-candidate comparison use seed 2026, `checkpoint_best_ema.pth`, selected by the highest validation Mask AP. Three-seed summaries were obtained from the corresponding best EMA checkpoint for each seed. Aggregate statistics are available for all three seeds, whereas detailed run artifacts needed for paired seed-level reanalysis are available only for the primary seed-2026 experiment. The final held-out-test operating protocol was fixed before evaluation.
Training batch configuration: Batch size 4 with 7× gradient accumulation (effective batch size 28) for the repeated RF-DETR-based training configurations.
Inference hardware and timing protocol: The runtime used an NVIDIA RTX 4090, CUDA 12.1, and PyTorch 2.4, with mixed precision disabled for timing and batch size 1. Timing values and configuration labels are reported in the main efficiency table. **Experimental registry:** Table SI distinguishes the frozen core, fixed-candidate comparison, training-seed summaries, and refined output. The 20% core uses corrected references, 768/614 tiling, confidence 0.20, box-IoU threshold 0.5, and mask-IoM threshold 0.6. The table distinguishes single-run results from three-seed summaries and separates core from refined outputs.

Patch AP is computed on the validation-patch split before drawing-level consolidation. The patch-generation record contains 1,228 patches: 788 with annotations and 440 negative patches. Each patch is 768 × 768 pixels, with stride 384 (50% overlap). Right and bottom terminal patches are shifted left/up to cover the complete drawing boundary; if a drawing is smaller than 768 pixels, the uncovered right/bottom area is white-padded. This is not symmetric four-sided padding. Box AP uses box IoU thresholds from 0.50 to 0.95 in increments of 0.05; Mask AP uses the corresponding mask-IoU thresholds; AP_{50} and AP_{75} use fixed IoU thresholds of 0.50 and 0.75. These AP quantities are distinct from the class-aware mask-IoU ≥ 0.5 matching used for drawing-level Precision, Recall, false discovery rate (FDR), and F1. The evaluator uses confidence-ranked predictions with `maxDets`=500. Because RF-DETR

Table SI. Experimental result registry: checkpoints, statistical summaries, and evaluated outputs. Single-run seed-2026 outputs are distinguished from three-seed aggregate summaries.

Result object	Checkpoint/seed	Statistics	Evaluated input and output
Frozen DSC core	seed 2026; `checkpoint_best_ema.pth`	single-run Micro/Macro; bootstrap	corrected test set; raw candidates → DSC consolidation
Consolidation comparison	seed 2026; same `checkpoint_best_ema.pth`	single-run operator comparison	identical candidate export; consolidation adapter only
Repeated-seed DSC core	seeds 2026–2028	mean ± sample SD	one validation-selected EMA checkpoint per seed; core output
Patch AP ablations	seeds 2026–2028	retained mean ± sample SD	local-recognition ablations on the common validation-patch split
Application refinement	refined output	mean ± sample SD across seeds 2026–2028	DSC core → application refinement

exports at most 200 query predictions per patch (`num_select=200`) before evaluation, this evaluator cap is non-binding. No fixed 0.20 confidence pre-filter is used for Patch AP; 0.20 is reserved for full-diagram candidate export and DSC consolidation. The reported local-recognition ablations use the same 1,228 validation patches, including the same 440 negative patches and corrected annotation version. Three-seed aggregate summaries are reported separately from single-run diagnostics; detailed run artifacts needed for paired seed-level reanalysis are available only for the primary seed-2026 experiment. The reported protocol does not apply a separate 5% visible-area ignore rule.

Patch latency: Reported as the median over 100 timed forward passes on 768×768 inputs, preceded by 10 warm-up forward passes. Explicit GPU synchronization is invoked before and after each timed pass. **Full-diagram time:** The timing log reports a 3.7 s median over the 10 held-out test diagrams for the full application pipeline. It includes patch generation, model forward, drawing-coordinate projection, class-wise NMS, mask-containment suppression, OCR, and railway-rule refinement. Parameter counts in the efficiency table refer to the local segmentation model only and exclude PaddleOCR and rule code. DXF rendering and disk I/O are excluded, as in the main timing definition.

Overlap selection and test sensitivity. Nominal overlaps of 0%, 20%, 25%, and 50% were compared on the fourteen validation drawings using the same checkpoint, confidence cutoff (0.20), box-NMS threshold (0.5), mask-IoM threshold (0.6), class vocabulary, and drawing-level evaluator. Only tile overlap was varied. The evaluated output was the consolidated DSC-core instance set after confidence filtering, projection, and DSC consolidation, before OCR or railway-rule refinement. The primary selection criterion was validation drawing-level Micro F1 computed from pooled counts. Exact ties were resolved first by higher Micro Recall and then by lower Micro FDR, using metrics before rounding. This procedure selected nominal 20% overlap with patch size 768 and stride 614; the regular-grid overlap is $1 - 614/768 \approx 20.05\%$. Terminal boundary alignment follows the same protocol described in the main paper. The selected overlap, stride, and all consolidation parameters were frozen before the subsequent method comparisons and held-out test evaluation. Test drawings were not used to select overlap or tune parameters. A post-selection sensitivity analysis was additionally performed on the held-out test drawings across the same four overlaps to quantify robustness, with the checkpoint and consolidation parameters held fixed; this analysis did not alter the validation-selected 20% operating point. This drawing-level selection procedure is distinct from the fixed 50% validation-patch split used for local AP. The mask-IoM threshold of 0.6 is recorded as selected on validation drawings using a recall–FDR trade-off. The per-setting validation counts and exact threshold-selection objective are not available in this supplement; the post-selection test grid does not reconstruct that selection record. The approximately 1.03 pp recall decrease relative to box NMS

in the held-out fixed-candidate comparison is reported as a test-set consequence of that validation-selected operating point, not as a threshold-selection criterion.

S-VII. Baseline Adaptation Details

The source methods for the adapters below are Spatial Mask Merging by Mihajlovic and Marjanovic (2025) and Remote SAMsing by de Carvalho et al. (2026), listed as References [13] and [16], respectively, in the main article. All evaluated adapters use the same projected candidate contract within each comparison protocol. DSC consolidation performs box NMS followed by mask-IoM suppression. The Spatial Mask Merging (SMM) adapter performs class-wise graph/ILP grouping and mask union. The overlapping-tile SAM-Mosaic adapter applies best-match association using shared foreground pixels within source-patch intersections, whereas the separate useful-area adapter uses fixed-boundary label merging. The group definitions below distinguish these operations; Algorithm S4 summarizes the overlapping comparison. These are adaptations to the common railway candidate generator, not comparisons of complete independently trained recognition systems.

Common input and adapter-specific output. In the overlapping comparison, the adapters replace their original candidate-generation stage with the same railway-labelled patch-predictor outputs, projected into drawing coordinates. SAM-Mosaic therefore receives neither SAM2-generated masks nor the separately evaluated non-overlapping useful-area crops. Candidate classes and the input confidence cutoff are shared; the output geometry and scores follow each adapter's recorded rule. For a merge group G, both adapters output the mask union $m_G = \bigcup_{i \in G} m_i$ and its tight bounding box. SMM assigns $s_G = |G|^{-1} \sum_{i \in G} s_i$, whereas SAM-Mosaic assigns $s_G = \max_{i \in G} s_i$. Neither adapter applies additional DSC consolidation. **SMM group construction.** Let V be the candidate indices of one predicted class, with box b_i, binary mask m_i, and confidence s_i. Spatial retrieval uses an R-tree to accelerate neighborhood search; the exact Euclidean box-distance test determines the spatial pair set

$$E_\rho = \{(i,j) : i,j \in V,\ i < j,\ d_{\text{box}}(i,j) \le \rho\}, \tag{S2}$$

where $d_{\text{box}}(i,j) = \min_{u \in b_i, v \in b_j} \|u - v\|_2$ and $\rho = 39$ pixels. R-tree retrieval does not replace this exact test. For each spatial pair, define the minimum distance between mask-boundary pixels and the mask IoU as

$$\begin{aligned} D_{ij} &= \min_{u \in \partial m_i,\, v \in \partial m_j} \|u - v\|_2, \\ I_{ij} &= \frac{|m_i \cap m_j|}{|m_i \cup m_j|}. \end{aligned} \tag{S3}$$

The pair weight is

$$\begin{aligned} w_{ij} = {} & \beta_1 \max(0, 1 - D_{ij}/\tau_d) \\ & + \beta_2 I_{ij} + \beta_3 \min(s_i, s_j). \end{aligned} \tag{S4}$$

The compatible pair set is

$$\begin{aligned} \mathcal{C} = \{(i,j) \in E_\rho : (D_{ij} \le \tau_d \text{ or } I_{ij} \ge \tau_i) \\ \text{and } w_{ij} \ge \gamma\}. \end{aligned} \tag{S5}$$

The corrected implementation adds the within-group all-pairs compatibility requirement. Let $\widehat{C}_{ij}$ denote the full pair-compatibility decision for candidates i and j under the configured geometric and weight tests. This decision is checked for every pair in a proposed group,

including pairs not directly joined during spatial graph construction; absence of an edge is not automatic evidence of compatibility. For a partition $\mathcal{P}$ of the class candidates, define $x_{ij}(\mathcal{P}) = 0$ if i and j are in the same group and 1 otherwise. At the partition level, the objective is

$$\min_{\mathcal{P}} \sum_{(i,j)\in E_\rho} \left[w_{ij}x_{ij}(\mathcal{P}) + \lambda(1-w_{ij})(1-x_{ij}(\mathcal{P}))\right], \tag{S6}$$

subject to the group-validity condition

$$\widehat{C}_{ij} = 1 \quad \text{for every } G \in \mathcal{P} \text{ and every } i,j \in G,\ i<j. \tag{S7}$$

The key condition is on all group members, rather than only the links of a connecting path. Thus, compatible links (i,j) and (j,k) cannot justify a common group when (i,k) is incompatible. An exhaustive final-output audit enumerates all unordered member pairs and computes

$$N_{\text{incompat}} = \sum_{G\in\mathcal{P}} \sum_{\substack{i,j\in G\\ i<j}} (1-\widehat{C}_{ij}). \tag{S8}$$

The corrected overlapping-comparison runs all returned $N_{\text{incompat}} = 0$. Section S-XIX reports their solver-status counts. These partition-level conditions describe the enforced requirement and final-output check; they do not prescribe an unrecorded auxiliary-variable or constraint-generation implementation. Each group produces its mask union, tight bounding box, and mean input confidence, with no additional DSC mask-IoM suppression. All reported overlapping runs use this corrected group-validity requirement.

Table SII. Parameters of the evaluated SMM graph/ILP adapter.

Parameter	Value	Role and unit
τ_d	6	Boundary-distance scale and cutoff (pixels)
τ_i	0.859	Mask-IoU compatibility threshold (dimensionless)
ρ	39	Exact box-distance cutoff (pixels)
β_1	0.362	Boundary-proximity weight (dimensionless)
β_2	0.461	Mask-IoU weight (dimensionless)
β_3	0.120	Confidence weight (dimensionless)
λ	0.777	Merge-cost coefficient in the ILP (dimensionless)
γ	0.476	Minimum compatible-pair weight (dimensionless)

Table SII gives the anchor settings for the corrected SMM adapter. The 20-setting sweep in Section S-XVIII varies γ and τ_d, keeping the other entries fixed and enforcing within-group all-pairs compatibility throughout. This is a descriptive test-set sweep rather than a validation-optimized baseline. DSC overlap and mask-IoM selection follow the validation procedure in Section S-VI.

SAM-Mosaic overlapping-tile group construction. The overlapping comparison uses `merge_sammosaic_overlap.py`. For source patch a, the metadata define its source-patch rectangle in drawing coordinates R_a by $(x_a, y_a, x_a + \text{width}_a, y_a + \text{height}_a)$. Terminal patches use their recorded actual rectangles with no additional matching rule. For candidate i, let $a(i)$ denote its source patch. A candidate pair is considered only if its classes agree, $a(i) \neq a(j)$, and the source-patch rectangles intersect. Its contact score is

$$\begin{aligned} R_{a(i),a(j)} &= R_{a(i)} \cap R_{a(j)}, \\ c_{ij} &= |m_i \cap m_j \cap R_{a(i),a(j)}|. \end{aligned} \tag{S9}$$

Contact is the count of shared foreground pixels inside the patch intersection; it is neither an IoU nor a mask-boundary distance. A pair is eligible when $c_{ij} \geq 1$ pixel. Each candidate independently selects the eligible partner with the greatest contact count. Ties select the

partner that appears first in the original candidate order. Mutual best-match agreement is not required: a selection in either direction adds the pair to the merge set. No additional confidence or IoU threshold is applied at this association stage; the common candidate-export confidence cutoff of 0.20 remains in force.

Union-Find converts the accepted pairs into connected components G. Unmatched candidates remain singleton groups. Each group produces the pixel-wise mask union $m_G = \bigcup_{i \in G} m_i$, its tight bounding box, and maximum input confidence $s_G = \max_{i \in G} s_i$. No additional DSC consolidation is applied. This overlap-contact rule is distinct from the fixed-boundary label merging in `sam_mosaic_merge_impl.py`, which is used for the separate non-overlapping useful-area protocol below; the two scripts do not define one common grouping rule.

The operator comparison evaluates complete DSC consolidation, SMM, and overlap-adapted SAM-Mosaic under fixed projected candidates. To isolate the two DSC consolidation stages, Direct, box-NMS-only, mask-IoM-only, and box-NMS-plus-box-IoS controls use the same pre-consolidation candidate export, confidence cutoff, class labels, initial candidate ordering, thresholds, and drawing-level evaluator. Each adapter then applies its own scoring, aggregation, and selection rule; in particular, SMM uses its mean-score aggregation and SAM-Mosaic uses its specified merge-score rule. None of these controls receives candidates already filtered by DSC consolidation.

The corrected SMM anchor row comes from the reported 20-setting scan: $TP = 1{,}697$, $FP = 793$, and $FN = 58$ at $(\gamma, \tau_d) = (0.476, 6)$. The measured SAM-Mosaic overlap sweep confirms $TP = 1{,}697$, $FP = 999$, and $FN = 58$ for bidirectional union best with one-pixel minimum contact (Section S-XX). The main comparison now uses these supplied integer counts rather than reconstructing them from rounded metrics.

Table SIII reports a non-overlapping useful-area protocol following the tiling and boundary-merging design of SAM-Mosaic (Remote SAMsing). It uses 768×768 useful areas on a ceil-sized grid, 50-pixel edge-replicated context padding, cropping back to each useful area, and confidence cutoff 0.40. The saved padded crop is 868×868 pixels; it is passed to the RF-DETR predictor, whose internal preprocessing handles the model input and returns boxes and masks in crop coordinates. Predictions are offset by the recorded crop origin, clipped to the 768×768 useful area, and merged. The overlapping comparison uses 768-pixel tiles, stride 614, and confidence cutoff 0.20. The non-overlapping and overlap SAM-Mosaic merge adapters are evaluated separately on the common predictor outputs for their respective protocols. Neither row denotes the complete SAM2-based Remote SAMsing pipeline. Results are not transferred between protocols.

Non-overlapping useful-area label construction. The native adapter in `sam_mosaic_merge_impl.py` constructs a separate integer label raster for each semantic class on a ceil-sized canvas. For a drawing of width W and height H, the canvas dimensions are $768\lceil W/768 \rceil$ and $768\lceil H/768 \rceil$. Candidates clipped to their useful areas are processed in descending confidence order. Each candidate writes its label only to currently unlabeled pixels of its foreground mask. Higher-confidence candidates therefore own shared pixels, while lower-confidence candidates occupy only the remaining pixels. A candidate whose entire mask is already occupied produces no effective label. Since useful-area tiles do not overlap, this rule resolves pixel conflicts within each useful area.

Native boundary best-match merging. Internal tile boundaries lie at grid coordinates $768, 1536, \ldots$. At each vertical boundary, the adapter compares labels in the immediately adjacent left and right columns; at each horizontal boundary, it compares the immediately adjacent upper and lower rows. It counts contacts for distinct, nonzero label pairs observed across these adjacent boundary pixels. Best-match selection is performed in both directions: each label selects the opposite-side label with the highest contact count. A pair selected

in either direction is accepted; mutual best-match agreement is not required. Union-Find converts the accepted pairs into connected groups, retaining unpaired effective labels as singletons. The output mask of a group is the union of the pixels assigned to its member labels in the raster, with a tight bounding box and the maximum confidence of those labels' source candidates. Thus, these native masks are formed from the confidence-resolved label raster.

The reported native configuration uses `best_match`. Every valid nonzero, distinct boundary-label pair participates in best-match selection. The `min_contact` argument does not impose an additional contact-count threshold in this branch; it is used for contact-count filtering only when `merge_strategy="min_contact"`. This native boundary procedure is separate from the shared-foreground contact definition for overlapping patches in Eq. S9.

Table SIII. Earlier non-overlapping useful-area comparison; all metrics are Micro. No application refinement is applied. The SMM row predates the corrected overlapping runs and is not covered by their all-pairs audit.

Merge method	Precision	Recall	FDR	F1
DSC	0.7265	0.8974	0.2735	0.8030
SMM (earlier adapter)	0.7209	0.8860	0.2791	0.7950
SAM-Mosaic merge adapter	**0.8469**	**0.9328**	**0.1531**	**0.8877**

The non-overlapping comparison includes all three operators under its stated candidate-generation protocol. SAM-Mosaic has the highest F1 in this setting, whereas DSC consolidation has the highest F1 in the overlapping comparison. Useful-area tiling, context padding, and confidence cutoff differ between settings; the ranking reversal is therefore interpreted as protocol-specific rather than as an isolated operator effect.

S-VIII. Repeated-Seed Full-Diagram Evaluation

The primary frozen result is the seed-2026 run loaded from `checkpoint_best_ema.pth`, selected by the highest validation Mask AP; the identical checkpoint and candidate export are used for the fixed-candidate operator comparison. For seeds 2026–2028, the corresponding best validation-selected EMA checkpoints are evaluated separately on all ten corrected test drawings under the fixed 20% protocol and summarized as mean $\pm$ sample SD; predictions are never pooled across seeds. The main-paper reliability table reports the core summaries, and its refined-output table reports the refined-output summaries. The frozen-checkpoint Macro P/FDR (0.8967/0.1033) differ from the core seed means (0.8966/0.1034). Only aggregate seed summaries are retained, preventing reconstruction of paired-effect intervals or seed-specific drawing bootstraps.

Training-seed SD describes checkpoint variation; the following frozen-core bootstrap describes drawing-sampling variation.

Local-recognition ablations and their three-seed summaries are reported in the main article. Class-specific Copy–Paste, representation, and centroid diagnostics remain single-run analyses.

S-IX. Diagram-Level Bootstrap Confidence Intervals

To characterize uncertainty caused by the limited number of held-out diagrams, complete diagrams are resampled with replacement; patches and individual instances are never resampled independently. The main-paper DSC-core intervals are recomputed directly from the ten per-drawing rows in Table SIX, using the authoritative pooled counts (GT=1,755, TP=1,662, FP=186, FN=93, predictions=1,848). We use NumPy's PCG64 generator via `numpy.random.default_rng(seed=2026)`, draw 10,000 samples of ten diagram IDs with replacement, recompute each metric for every resample, and report the two-sided 2.5th

and 97.5th percentiles (linear quantile interpolation). The core intervals are not transferred to the refined output.

Application summaries are reported as three-seed means with sample SDs in the main-paper refined-output table; diagram-level bootstrap intervals are reported separately for the frozen DSC core.

S-X. Residual Error Cases

Tables SV and SVI quantify residual errors and candidate-level suppression risks for the frozen seed-2026 core. FP and FN are first determined by class-aware greedy matching at mask IoU ≥ 0.5. The subsequent audit assigns descriptive association categories to these unmatched outputs. The different-class overlap category combines 21 entries originally labelled partial overlap and 19 labelled strong overlap, because a reproducible numerical boundary between those labels was not retained. The merged count is 40; the FP total remains 186. Association thresholds and precedence among competing error labels are not fully specified in the retained audit, so these counts describe recorded associations rather than an independently executable causal taxonomy. Table SIV provides plausible contributing mechanisms without treating them as verified causes.

Table SIV. Representative residual error types of the DSC core.

Error type	Plausible contributing factor	Affected output
Boundary omission	Severe truncation when no overlapping tile provides a sufficiently complete observation	Missing or incomplete instance mask
Small-object instability	Tiny joint symbols near dense structures or text annotations	Localization jitter
Track/switch confusion	Similar local appearance without topology-level context	Class ambiguity
Text interference	Dense text close to symbols or overlapping graphical elements	False-positive instance prediction

These residual cases motivate engineering review. The application refinement addresses a subset of application-specific errors after consolidation; its three-seed measurements are reported separately from the frozen core result. No rule-specific effect or refined-output bootstrap interval is inferred from the available aggregate summaries.

Table SV. Descriptive residual-error tally for the frozen DSC core. Percentages use the FP or FN total separately; the labels describe association patterns rather than causal mechanisms.

Type	Recorded category	Count	Within-type share (%)
FP	No annotated-target association	75	40.32
FP	Same-class mask overlap insufficient	68	36.56
FP	Overlap with a different-class target	40	21.51
FP	Same-class duplicate	3	1.61
FP total	–	186	100.00
FN	Same-class mask overlap insufficient	75	80.65
FN	No associated final prediction	11	11.83
FN	Wrong-class prediction	7	7.53
FN	One-to-one matching conflict	0	0.00
FN total	–	93	100.00

A potential-risk event is a suppressed same-class candidate whose IoU with a GT instance reaches 0.5 while the corresponding keeper does not. Multiple candidates can refer to one GT, and another survivor can still match that GT. The 120 flagged candidates out of 1,512 suppressions (7.94%) must therefore not be equated with 120 unique false negatives. The removal total equals $3,360 - 1,848 = 1,512$, the difference between the box-NMS and DSC output counts. The measured class-wise before/after tally identifies 18 GT instances that lose a match after mask-IoM. Their total equals the TP decrease from 1,680 after box

Table SVI. Class-wise residual errors, second-stage suppression audit, and measured GT match losses after mask-IoM. The loss column counts previously matched GT instances that become unmatched when mask-IoM is applied after box NMS. Risk percentages divide potential-risk candidates by suppressed candidates within each class. A dash denotes a zero denominator. Candidate risks do not count unique missed GT instances.

Class	FP	FN	Suppressed	Potential risk	Lost GT	Risk (%)
Signal	25	11	108	0	0	0.00
Text	49	26	124	17	2	13.71
Joint	29	15	87	0	0	0.00
Switch	15	7	33	4	1	12.12
Signal tower	1	0	0	0	0	–
Track section	12	6	298	37	5	12.42
Switch section	40	20	787	58	9	7.37
Fouling Point Indicator	11	5	66	4	1	6.06
Carrier Frequency	2	2	9	0	0	0.00
Repeater Signal	2	1	0	0	0	–
Total	186	93	1512	120	18	7.94

NMS to 1,662 after DSC. Under unchanged candidate geometry, scores, and matching order, this deletion-only step cannot create a match for a previously unmatched GT; 18 is consequently the loss total, while 120 counts candidate-level risks and is not an additional FN count. The largest match losses are nine switch sections and five track sections. The per-GT identity trace is not included in the available summary.

S-XI. Full Class-Wise Localization Residual Dispersion

Table SVII summarizes matched patch-level centroid residuals from one recorded DSC local-recognition run: seed 2026 with `checkpoint_best_ema.pth`. It uses the common 1,228-patch validation split (788 annotated and 440 negative patches), class-aware greedy matching at mask IoU $\geq$ 0.5, and the common patch evaluator before drawing-level consolidation. The 14,645 matched pairs are from this specified run only; no pooling across seeds or averaging of residual moments was performed. Counts are observations rather than independent drawing instances.

Table SVII. Full class-wise patch-level localization residual dispersion (Level 1, before document-space consolidation). n_{TP} denotes the number of matched patch-level prediction–reference pairs per class; the same full-diagram instance may contribute multiple pairs across overlapping patches. Unit: pixels.

Category	n_{TP}	$\bar{\Delta c_x}$	$\bar{\Delta c_y}$	s_{c_x}	s_{c_y}
Overall	14645	−0.03	−0.08	2.69	1.22
signal	1531	−0.15	−0.29	1.56	1.20
text	3356	−0.35	−0.14	0.91	0.87
joint	2149	−0.25	0.01	0.78	0.81
switch	1178	−0.17	−0.17	1.31	0.74
signal tower	42	−0.04	−0.04	0.98	0.92
track section	1497	0.56	−0.03	5.58	0.72
switch section	3803	0.33	0.02	3.48	1.82
Fouling Point Indicator	812	−0.38	−0.04	1.53	1.23
Carrier Frequency	185	−0.29	−0.31	0.62	0.88
Repeater Signal	92	0.09	−0.34	0.89	0.71

S-XII. Source-Stratified and Per-Drawing Results

The held-out test set contains six same-source hold-out diagrams and four preliminary unseen-source diagrams. For this anonymized analysis, D1, D2, D5, and D6 are assigned to the unseen-source stratum; the remaining six diagrams form the seen-source stratum. Micro metrics are computed from pooled counts, whereas Macro metrics are arithmetic means of the corresponding per-drawing metrics.

Quantitative error and candidate-risk tallies, together with the limits of their attribution, are given in Section S-X.

Table SVIII. Source-stratified DSC-core results under the locked 20% protocol.

Subset	Drawings	GT	TP	FP	FN	Micro P	Micro R	Micro FDR	Micro F1	Macro P	Macro R	Macro FDR	Macro F1
Seen-source	6	1,231	1,162	126	69	0.9022	0.9439	0.0978	0.9226	0.9001	0.9504	0.0999	0.9245
Unseen-source	4	524	500	60	24	0.8929	0.9542	0.1071	0.9225	0.8916	0.9561	0.1084	0.9227
Overall	10	1,755	1,662	186	93	0.8994	0.9470	0.1006	0.9226	0.8967	0.9527	0.1033	0.9238

Table SIX. Per-drawing DSC-core document-space results under the locked 20% protocol; D1–D10 are anonymized identifiers.

Diagram	GT	TP	FP	FN	Pred.	Precision	Recall	FDR	F1
D1	212	201	23	11	224	0.8973	0.9481	0.1027	0.9220
D2	120	115	14	5	129	0.8915	0.9583	0.1085	0.9237
D3	326	304	31	22	335	0.9075	0.9325	0.0925	0.9198
D4	104	100	12	4	112	0.8929	0.9615	0.1071	0.9259
D5	83	80	10	3	90	0.8889	0.9639	0.1111	0.9249
D6	109	104	13	5	117	0.8889	0.9541	0.1111	0.9204
D7	113	109	12	4	121	0.9008	0.9646	0.0992	0.9316
D8	114	110	13	4	123	0.8943	0.9649	0.1057	0.9283
D9	259	244	27	15	271	0.9004	0.9421	0.0996	0.9208
D10	315	295	31	20	326	0.9049	0.9365	0.0951	0.9204
Micro	1755	1662	186	93	1848	0.8994	0.9470	0.1006	0.9226
Macro	–	–	–	–	–	0.8967	0.9527	0.1033	0.9238

S-XIII. Application Refinement: OCR and Railway Rules

The application refinement applies OCR and railway-specific rules to the DSC-core instance set. Its P/R/FDR/F1 summary is reported separately from the primary DSC-core evaluation as the mean across the three training-seed runs (2026–2028), with sample SDs in the main-paper refined-output table.

S-XIII.A. Enabled Refinement Rules

The application refinement consumes the consolidated prediction JSON without rerunning the detector or DSC consolidation. OCR is applied to text candidates covering the possible text annotations in the inventory; OCR recognition is followed by rule-based retention, rejection, or relabeling. The enabled decision sequence is:

1. Remove scores below 0.20. Crop each Text box with 12-pixel padding on all sides, clip to image boundaries, and run PaddleOCR (English) at 0/90/180/270 degrees; retain the highest-confidence orientation. If the original crop returns no text, retry a 2× bicubic-upsampled grayscale crop. If OCR remains empty, the enabled `-keep-ocr-fail` branch retains the original candidate: only `ocr_text` is set to an empty string, while `category_id`, `score`, `bbox`, `mask_rle`, and all other metadata remain unchanged. This candidate bypasses regex-based recognition, relabeling, and OCR-dependent rejection in the next step. Retention at the OCR stage does not bypass later enabled spatial rules; any applicable spatial rule is still evaluated and may subsequently relabel or remove the candidate. For nonempty OCR results, apply Unicode NFKC, uppercase conversion, whitespace removal, and slash/dash normalization.

2. For nonempty recognized strings, reject Chinese text. Carrier Frequency and numeric FPI candidates must fully match the corresponding patterns in Table SX. For numeric FPI candidates, Canny, HoughLinesP, and HoughCircles are applied in a crop expanded by 12 pixels; a line endpoint must satisfy

$$d(\text{endpoint}, \text{circle centre}) \leq r_{\text{circle}} + 30$$

in pixels. Remaining names use the regex-generated whitelist in the same table. Chinese text, measurements, bracketed numbers, invalid hyphenated strings, and non-matching prefixes are rejected. Accepted candidates remain Text or are relabeled Carrier Frequency/FPI. Existing Carrier Frequency/FPI candidates are not OCR-validated in this run.
3. Apply `-refine-section-classes` and `-drop-unconnected-sections`. Skeletonize each section mask using `skimage.morphology.skeletonize`, with a Zhang–Suen fallback. Select degree-1 skeleton endpoints; for branched skeletons, use the two endpoints with maximum Euclidean separation, and use the two PCA-extreme points if fewer than two endpoints are available. For endpoint p and joint/switch box $B = [x_1, y_1, x_2, y_2]$, compute

$$\begin{aligned} \delta_x &= \max(x_1 - p_x, 0, p_x - x_2), \\ \delta_y &= \max(y_1 - p_y, 0, p_y - y_2), \\ d(p, B) &= \sqrt{\delta_x^2 + \delta_y^2}. \end{aligned}$$

A distance ≤ 35 pixels denotes connection. If both endpoints connect, assign Switch section when either node is a switch and Track section otherwise; if either fails, the original class is retained before the enabled deletion removes the unconnected section.

Text-isolation deletion (default anchor distance 350 pixels), generic isolation, FPI–signal overlap gating, and joint-connectivity processing (12-pixel dilation) are not enabled. They contribute no claimed effect to this configuration. Retained mask geometry is unchanged.

Table SX. OCR patterns used for nonempty recognized strings after normalization.

Candidate type	Pattern
Carrier Frequency	`^[0-9]+-[0-9]+(?:/[0-9]+-[0-9]+)?$`
Numeric FPI	`^[0-9]{3,4}(?:\.[0-9]+)?$`
F names	`^[F][A-Z][A-Z0-9]*$`
Section names	`^(?!G)(?=[A-Z0-9]{1,12}$)[A-Z0-9]*G[A-Z0-9]*$`
Signal names	`^[XSJFDY](?:[A-Z]{0,3})?[0-9]{0,4}[A-Z]?$`
Switch numbers	`^[0-9]{1,2}$`

The OCR engine is PaddleOCR with English recognition. The fixed pixel distances refer to the evaluated raster scales and are not physical-distance tolerances.

The implementation writes `ocr_audit.csv`, `section_topology.csv`, and related records containing drawing and input-instance indices, decisions, proposed classes, endpoint nodes, and reasons. Candidate-to-GT transition records can explain the effect of individual rules; the overall refined-output metrics are computed by evaluating the complete refined output with the same class-aware matching rule.

S-XIV. Additional Recognition Results and Visualizations

The main-paper local-recognition ablations include the single-run Copy–Paste, representation-bundle, and compact-centroid diagnostics. This section provides additional per-category Box AP and qualitative overlays. These descriptive results use the patch protocol in Section S-VI and are distinct from three-seed aggregate summaries.

Table SXI. Per-category Box AP under different compensation configurations.

Config.	joint	track sec.	Carrier F.
Baseline + CP	0.4791	0.6830	0.6176
+Repr. comp.	0.4894	**0.7186**	0.6478
+Gated Wasserstein only	0.4797	0.6811	**0.6932**
+Repr. comp. + Gated Wasserstein	**0.5096**	0.7177	0.6869

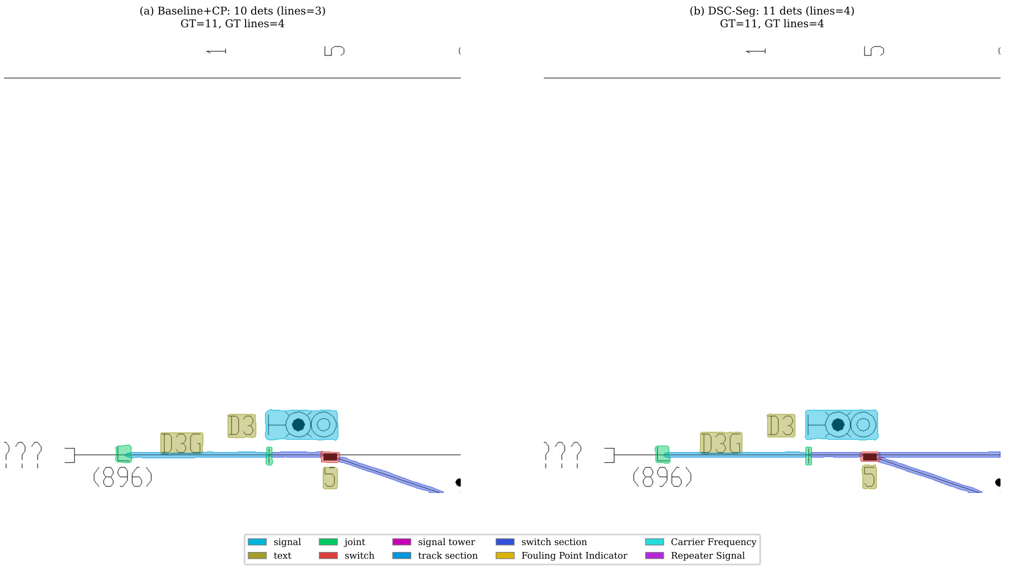

Figure S2. Baseline+CP and DSC on a representative region. In the panel label, "DSC-Seg" denotes the DSC local-recognition configuration used for this illustration and is not a separate method family. This comparison shows the combined local-recognition and selection pipeline, not an isolated effect of one component.

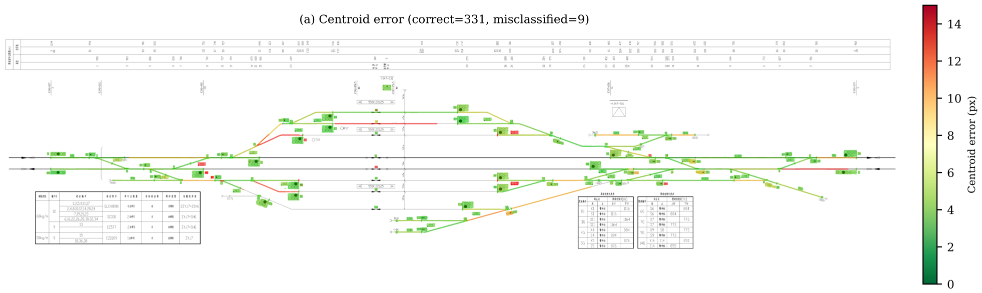

Figure S3. Errors in the full-drawing overlay obtained by directly projecting patch predictions to their drawing coordinates, before box NMS, mask-IoM suppression, or application refinement. The color bar indicates centroid error in pixels; the correct/misclassified labels describe this illustrative output rather than the pooled held-out evaluation. The errors remaining after coordinate projection alone motivate drawing-level post-processing. Quantitative consolidation effects are reported in the main-paper fixed-candidate comparison.

S-XV. Reproducibility Algorithms

The source diagrams, annotations, production checkpoints, and production code are proprietary. To make the method auditable without releasing protected material, this section specifies the reported core operations, evaluation procedure, and available baseline-adapter parameters. Core consolidation is deterministic for a fixed candidate list, thresholds, and original order.

Algorithm S1 Complete DSC inference on one full-resolution diagram

Require: Raster diagram I; tile size $w = 768$; stride $q = 614$; score threshold $\tau_s = 0.20$; NMS threshold $\tau_b = 0.5$; IoM threshold $\tau_c = 0.6$
Ensure: Final document-space candidates $\mathcal{L}$

$\mathcal{D} \leftarrow [\,]$
$\mathcal{L} \leftarrow [\,]$
for tile origins generated by the fixed stride protocol, including a deduplicated terminal origin **do**
    Crop tile P at offset $o = (x, y)$; pad only the right/bottom when the diagram is smaller than w
    Run patch predictor: $(\hat{c}, \hat{b}, \hat{m}, s) \leftarrow h_\theta(P)$
    for each prediction with $s \geq \tau_s$ **do**
        Use the detector-emitted binary mask; discard empty masks; translate the box by (x, y, x, y) and place the mask by a two-dimensional pixel translation; clip both to diagram bounds and discard any mask that becomes empty; append $(\hat{c}, \hat{b}, \hat{m}, s, \text{tile-id}, o)$ to $\mathcal{D}$
    end for
end for
Group $\mathcal{D}$ by predicted class
for each class group **do**
    $\mathcal{K} \leftarrow [\,]$
    Sort by descending s, then ascending original index
    Apply class-wise box NMS at τ_b
    Preserve this priority sequence for the IoM scan
    Apply Algorithm S2 to the NMS survivors at τ_c, obtaining $\mathcal{K}$
    $\mathcal{L} \leftarrow \mathcal{L} \,\|\, \mathcal{K}$
end for
return concatenation of retained class groups $\mathcal{L}$

Algorithm S2 MaskIoMSuppress: class-wise mask-containment suppression after box NMS

Require: Same-class candidates (b_i, m_i, s_i) in drawing coordinates after class-wise box NMS; $\tau_c = 0.6$
Ensure: Retained list $\mathcal{L}$

$\mathcal{L} \leftarrow [\,]$
Sort by descending s_i, then ascending original index; preserve this order
for each candidate i in priority order **do**
    covered $\leftarrow$ false
    for each already retained candidate $k \in \mathcal{L}$ **do**
        if $\text{IoM}(m_i, m_k) = |m_i \cap m_k| / \min(|m_i|, |m_k|) > \tau_c$ **then**
            covered $\leftarrow$ true; **break**
        end if
    end for
    if not covered **then**
        append i to $\mathcal{L}$
    else
        suppress i
    end if
end for
return $\mathcal{L}$

DSC consolidation denotes the complete two-stage containment-aware operator. Its second-stage subroutine is named MASKIOMSUPPRESS and is applied after class-wise box NMS; only same-class NMS survivors enter this subroutine. DSC consolidation uses no tile provenance, boundary-contact flag, truncation direction, completeness score, mask fusion, fragment stitching, or topology reconstruction. The retained candidate is copied unchanged.

Algorithm S3 Evaluation, matching, macro/micro metrics, and diagram bootstrap

Require: Per-diagram predictions and GT masks; matching IoU $\tau_m = 0.5$; bootstrap replicates $B = 10000$; PCG64 seed 2026

1: **for** each diagram **do**
2: Group predictions and GT by class; sort predictions by descending confidence, breaking equal scores by ascending original candidate index
3: **for** each prediction **do**
4: Match to the unmatched GT of the same class with maximum mask IoU if IoU $\geq \tau_m$; ties in maximum IoU use ascending GT index; otherwise label FP
5: **end for**
6: Label each unmatched GT as FN; every unmatched prediction is FP; record $(TP_d, FP_d, FN_d, GT_d, Pred_d)$
7: **end for**
8: For the reported ten-drawing dataset, $GT_d > 0$ and $Pred_d > 0$: $P_d = TP_d/Pred_d$, $R_d = TP_d/GT_d$, $FDR_d = FP_d/Pred_d$, $F1_d = 2TP_d/(2TP_d + FP_d + FN_d)$
9: Macro metrics $\leftarrow$ arithmetic mean over all evaluated diagrams on the stated nonempty drawing-level evaluation set
10: Micro metrics $\leftarrow$ recompute from pooled $TP = \sum TP_d$, $FP = \sum FP_d$, $GT = \sum GT_d$, $Pred = \sum Pred_d$
11: **for** $b = 1, \ldots, B$ **do**
12: Sample n complete diagrams with replacement from the n evaluated diagrams
13: Recompute the four micro metrics from pooled counts and the four macro metrics as arithmetic means of the resampled per-diagram metrics
14: **end for**
15: Use the 2.5th and 97.5th percentiles with the implementation's linear quantile interpolation
16: **return** Point estimates and percentile 95% intervals from the B bootstrap replicates

Algorithm S4 Fixed-candidate SMM and SAM-Mosaic overlap adapters

Require: Common projected candidates after score cutoff 0.20, including class, score, box, mask, source-patch metadata and original index

1: **Corrected SMM:** group candidates by class; retrieve spatial pairs using the exact box-distance cutoff $\rho = 39$
2: Compute boundary distance, mask IoU, and pair weights using Eq. S4
3: Optimize the grouping with the all-pairs validity requirement in Eqs. S6–S7; record solver status and time limit
4: Enumerate every unordered pair in each final group, including pairs without a direct graph edge; count compatibility violations
5: Require $N_{\text{incompat}} = 0$; report any failure rather than accepting a connected but incompatible group
6: Output each group's mask union, tight box, and mean input score
7: **SAM-Mosaic overlap:** enumerate same-class candidate pairs from different intersecting source-patch rectangles
8: Compute contact using Eq. S9; keep pairs with at least one shared foreground pixel
9: Each candidate chooses its greatest-contact partner; ties use original partner order; mutual agreement is not required
10: Apply Union-Find to accepted pairs, retaining unmatched candidates as singletons
11: Output each SAM-Mosaic group's mask union, tight box and maximum input score
12: Apply no additional DSC consolidation to either output; evaluate with the common class-aware mask-IoU matching on the same drawings

S-XVI. Data and Code Availability

The source drawings, corrected annotations, production checkpoints, and production inference code are unavailable for public distribution under proprietary railway-project restrictions. No authorized anonymized subset or validated synthetic dataset is available. The manuscript and Supplementary Material contain the reported protocols, pseudocode, threshold analyses, and anonymized aggregate and per-drawing results. These materials support procedural transparency but do not enable independent reproduction of the private-data numerical results.

S-XVII. Joint Threshold Sensitivity

The DSC threshold sweep uses the frozen seed-2026 checkpoint and the same pre-consolidation candidates, corrected references, 20% overlap, confidence cutoff 0.20, class vocabulary, and greedy evaluator as the main comparison. Only box-NMS and mask-IoM thresholds change. The 25 test-set combinations are post-selection diagnostics and do not change the validation-selected $(0.50, 0.60)$ operating point. The full grid reports Micro F1; the two operating-point slices also report TP/FP/FN, precision, recall, and FDR. These pooled values underlie the main-paper threshold curves. The error and suppression audit in Section S-X describes the fixed operating point; the measured threshold responses for three selected classes and pooled counts for the remaining seven classes are supplied separately in Section S-XXI.

Table SXII. One-dimensional threshold slices on the ten test drawings with fixed candidates. A dagger identifies the validation-selected operating value; it does not denote a new test-set selection.

Varied threshold	Value	TP	FP	FN	P	R	F1	FDR
τ_b; $\tau_c = 0.60$	0.30	1660	201	95	0.8920	0.9459	0.9181	0.1080
τ_b; $\tau_c = 0.60$	0.40	1661	208	94	0.8887	0.9464	0.9167	0.1113
τ_b; $\tau_c = 0.60$	$0.50^{\dagger}$	1662	186	93	0.8994	0.9470	0.9226	0.1006
τ_b; $\tau_c = 0.60$	0.60	1665	212	90	0.8871	0.9487	0.9169	0.1129
τ_b; $\tau_c = 0.60$	0.70	1665	214	90	0.8861	0.9487	0.9163	0.1139
τ_c; $\tau_b = 0.50$	0.50	1664	210	91	0.8879	0.9481	0.9171	0.1121
τ_c; $\tau_b = 0.50$	$0.60^{\dagger}$	1662	186	93	0.8994	0.9470	0.9226	0.1006
τ_c; $\tau_b = 0.50$	0.70	1665	232	90	0.8777	0.9487	0.9118	0.1223
τ_c; $\tau_b = 0.50$	0.80	1669	262	86	0.8643	0.9510	0.9056	0.1357
τ_c; $\tau_b = 0.50$	0.85	1672	284	83	0.8548	0.9527	0.9011	0.1452

Table SXIII. Micro F1 for all threshold combinations. Rows vary box-IoU and columns vary mask-IoM. The dagger marks the frozen operating point.

$\tau_b \backslash \tau_c$	0.50	0.60	0.70	0.80	0.85
0.30	0.9114	0.9181	0.9150	0.9111	0.9086
0.40	0.9221	0.9167	0.9123	0.9070	0.9045
0.50	0.9171	$0.9226^{\dagger}$	0.9118	0.9056	0.9011
0.60	0.9134	0.9169	0.9110	0.9035	0.8979
0.70	0.9127	0.9163	0.9108	0.9021	0.8946

S-XVIII. Expanded SMM Parameter Evaluation

The sweep varies the compatibility-weight cutoff γ and boundary-distance scale τ_d using fixed candidates. Every setting uses the corrected adapter with within-group all-pairs compatibility. The scan does not re-optimize the remaining SMM coefficients or modify the evaluation protocol. The largest F1 is 0.7999, reached at $\gamma = 0.476$ with $\tau_d = 9$ or 12 pixels, compared with 0.7995 for the original setting. Increasing γ to 0.650 produces recall 0.9772 but precision about 0.616 and F1 about 0.755, demonstrating that the higher retained recall comes with many additional false positives. All counts satisfy $TP + FN = 1,755$.

Table SXIV. Twenty corrected SMM adapter settings on fixed candidates. The dagger marks the anchor parameter configuration, not the earlier implementation. Other SMM parameters retain the values in Table SII. The varied parameters belong to SMM; this table is separate from the DSC threshold grid and does not constitute a SAM-Mosaic parameter sweep.

γ	τ_d (px)	TP	FP	FN	P	R	FDR	F1
0.300	3	1605	794	150	0.6690	0.9145	0.3310	0.7727
0.300	6	1601	796	154	0.6679	0.9123	0.3321	0.7712
0.300	9	1598	797	157	0.6672	0.9105	0.3328	0.7701
0.300	12	1585	803	170	0.6637	0.9031	0.3363	0.7651
0.400	3	1613	792	142	0.6707	0.9191	0.3293	0.7755
0.400	6	1605	794	150	0.6690	0.9145	0.3310	0.7727
0.400	9	1601	796	154	0.6679	0.9123	0.3321	0.7712
0.400	12	1590	801	165	0.6650	0.9060	0.3350	0.7670
0.476	3	1699	795	56	0.6812	0.9681	0.3188	0.7997
0.476†	6	1697	793	58	0.6815	0.9670	0.3185	0.7995
0.476	9	1697	791	58	0.6821	0.9670	0.3179	0.7999
0.476	12	1697	791	58	0.6821	0.9670	0.3179	0.7999
0.550	3	1704	899	51	0.6546	0.9709	0.3454	0.7820
0.550	6	1704	897	51	0.6551	0.9709	0.3449	0.7824
0.550	9	1704	897	51	0.6551	0.9709	0.3449	0.7824
0.550	12	1704	896	51	0.6554	0.9709	0.3446	0.7825
0.650	3	1715	1071	40	0.6156	0.9772	0.3844	0.7553
0.650	6	1715	1069	40	0.6160	0.9772	0.3840	0.7557
0.650	9	1715	1069	40	0.6160	0.9772	0.3840	0.7557
0.650	12	1715	1069	40	0.6160	0.9772	0.3840	0.7557

S-XIX. Consolidation Resource Accounting

Each timing repetition processes all ten held-out drawings. For each method and stage, the per-drawing times are first summarized by a median within that repetition. Table SXV then summarizes these five repetition medians: its SD is the sample standard deviation with denominator four, and its IQR is the interquartile range across repetitions. These are not statistics pooled across all 50 drawing runs. Core and merge times are measured separately, not obtained by adding stage medians. The separate 3.7 s full-application result includes OCR and railway rules.

Table SXV. Timing summaries across five repetition-level per-drawing medians. All time columns are in seconds per drawing. The reported six-decimal summaries use the underlying timing records.

Stage	Adapter	Repeats	Median	Sample SD	IQR
Merge	Box NMS + box-IoS	5	0.010608	0.000130	0.000067
Merge	DSC	5	0.712726	0.014718	0.001995
Merge	SMM (corrected)	5	4.870839	0.041179	0.014326
Merge	SAM-Mosaic (overlap)	5	0.949160	0.103381	0.180992
Core	Box NMS + box-IoS	5	1.219570	0.021575	0.018067
Core	DSC	5	1.872373	0.040277	0.038045
Core	SMM (corrected)	5	5.922665	0.270537	0.274692
Core	SAM-Mosaic (overlap)	5	1.896799	0.137013	0.209834

Table SXVI. Core-pipeline per-drawing median for each repetition over ten drawings (seconds per drawing). Values are displayed to five decimal places; the more precise summaries above differ slightly from recalculations using these rounded values.

Adapter	Repeat 1	Repeat 2	Repeat 3	Repeat 4	Repeat 5
Box NMS + box-IoS	1.21957	1.26338	1.21110	1.23020	1.21214
DSC	1.96004	1.86533	1.90546	1.86741	1.87237
SMM (corrected)	6.46204	5.79029	6.15691	5.88222	5.92266
SAM-Mosaic (overlap)	2.06621	1.84607	2.15012	1.89680	1.85638

The corrected SMM status summary in Table SXVII covers the 20-setting scan and the timing runs. Each recorded scan run corresponds to one parameter setting and one drawing;

each formal timing scope has five repetitions of ten drawings. All recorded statuses are optimal, with zero timeouts and zero unknown timeout statuses. The exhaustive final-group compatibility audit includes every unordered member pair, irrespective of direct graph adjacency. The status counts describe these recorded runs; they do not enumerate any internal subproblem calls.

Table SXVII. Recorded solver status and final-group compatibility audit for corrected SMM. Warm-up is excluded from formal timing summaries.

Scope	Runs	Optimal	Timeout	Timeout status unknown	Incompatible pairs
20-setting scan	200	200	0	0	0
Formal merge timing	50	50	0	0	0
Formal core timing	50	50	0	0	0
Merge warm-up	1	1	0	0	0

Reported single-solve times in the parameter scan have minimum 0.00729 s, median 0.02972 s, and maximum 0.40949 s, with a 300 s limit per solve. Solver time and complete adapter merge time are distinct scopes: the latter can also include pair construction, geometric calculations, and mask-output construction. The timing record does not separately measure those components, so their costs are not obtained by subtracting medians. Optimal status refers to the encoded model; the separate all-pairs output audit establishes compatibility of the returned groups in these runs.

The rented instance uses an AMD EPYC 7K62 48-Core Processor host, 24 allocated CPU cores, platform-advertised system memory of 62G, and a platform-designated "4090-48G" GPU with advertised memory of 48G. These are reported capacities, not measured usage peaks. Earlier peak CPU RSS values were 2493, 2692, 3216, and 2476 MiB for box-IoS, DSC, the earlier SMM adapter, and SAM-Mosaic, respectively. They are separate earlier measurements; no corrected-SMM peak RSS is inferred from the earlier 3216 MiB value. Software thread settings, RSS sampling intervals and process scope, measured GPU peaks, and per-drawing timing traces are not available in the supplied record. The core repetition medians above quantify repeat variability, but cannot recover drawing-level timing distributions or establish a statistically significant DSC–SAM speed advantage.

S-XX. Controlled SAM-Mosaic Association Variants

The following 20 settings were measured using the same frozen seed-2026 candidates, corrected GT, confidence cutoff 0.20, 20% overlapping tiles, and evaluator as the main fixed-candidate comparison. Only the association strategy and contact threshold change. For contact c_{ij} defined in Equation S9, the eligible partner set is $E_t(i) = \{j : c_{ij} \geq t\}$ subject to the same-class, distinct-source-patch, and intersecting-patch requirements. The threshold t takes values 1, 5, 10, 25, and 50 shared foreground pixels.

For *one-way best*, source patches are sorted by $(y_{\min}, x_{\min}, y_{\max}, x_{\max}, \text{filename})$. Each candidate selects its maximum-contact eligible partner only from later patches in that spatial order. The maximum is taken across all eligible candidates in all later patches, not separately within each patch pair. For *bidirectional union best*, each candidate selects its maximum-contact partner across all of $E_t(i)$; an unordered edge is accepted if either endpoint selects the other. This is the original overlap adapter's rule. For *mutual best*, each candidate makes the same unrestricted selection, but an edge is accepted only when both endpoints select one another. For *all contact pairs*, every eligible unordered pair is accepted. The common candidate ordering resolves equal-contact ties as in Section S-VII.

Each strategy passes its accepted edges to the same Union-Find grouping and outputs the group mask union, tight box, and maximum input score. Candidate generation, masks, scores, and evaluation matching remain fixed. The strategies therefore control association

within one overlap protocol; they do not reproduce the complete SAM2-based Remote SAMsing system. With fixed contacts and tie order, raising the contact threshold only removes accepted edges: a maximum-contact partner remains the same whenever it is still eligible. Connected components can therefore split but cannot merge as this threshold increases. The resulting TP, FP, and F1 need not be monotone, because group masks and scores change.

Table SXVIII. Measured SAM-Mosaic overlap-adapter variants on the same frozen candidates and ten test drawings. Contact is the number of shared foreground pixels in the source-patch intersection, not a distance. The dagger marks the unchanged anchor setting.

Strategy	Contact	TP	FP	FN	P	R	F1
One-way best	1	1701	1000	54	0.6298	0.9692	0.7635
One-way best	5	1704	1002	51	0.6297	0.9709	0.7640
One-way best	10	1708	999	47	0.6310	0.9732	0.7656
One-way best	25	1711	1006	44	0.6297	0.9749	0.7652
One-way best	50	1712	1015	43	0.6278	0.9755	0.7639
Bidirectional union best	1†	1697	999	58	0.6295	0.9670	0.7625
Bidirectional union best	5	1701	1000	54	0.6298	0.9692	0.7635
Bidirectional union best	10	1702	1000	53	0.6299	0.9698	0.7637
Bidirectional union best	25	1702	1010	53	0.6276	0.9698	0.7620
Bidirectional union best	50	1705	1017	50	0.6264	0.9715	0.7617
Mutual best	1	1722	1676	33	0.5068	0.9812	0.6683
Mutual best	5	1722	1676	33	0.5068	0.9812	0.6683
Mutual best	10	1722	1676	33	0.5068	0.9812	0.6683
Mutual best	25	1722	1676	33	0.5068	0.9812	0.6683
Mutual best	50	1722	1682	33	0.5059	0.9812	0.6676
All contact pairs	1	1533	963	222	0.6142	0.8735	0.7212
All contact pairs	5	1550	967	205	0.6158	0.8832	0.7257
All contact pairs	10	1577	962	178	0.6211	0.8986	0.7345
All contact pairs	25	1608	968	147	0.6242	0.9162	0.7426
All contact pairs	50	1646	967	109	0.6299	0.9379	0.7537

The largest measured F1 values are 0.7656 for one-way best at 10 pixels, 0.7637 for bidirectional union best at 10 pixels, 0.7537 for all contact pairs at 50 pixels, and 0.6683 for mutual best at 1–25 pixels. The one-way maximum improves the anchor by approximately 0.31 percentage points, but remains approximately 15.70 percentage points below DSC (0.9226). Mutual best has high recall (0.9812) and many FP (1676–1682), showing that its conservative edge selection leaves a different output inventory. All-contact association has lower recall at small thresholds. These output metrics do not alone distinguish residual duplicates from wrong group associations; no causal error decomposition of SAM variants is inferred. The operating point used in the primary and timing comparisons remains bidirectional union best at one pixel. The test-set maximum is a diagnostic, not a replacement selected on held-out data.

S-XXI. Selected-Class Threshold Responses and Count Reconciliation

These measured slices resolve Text, Track Section, and Switch Section separately. Their GT counts are 487, 103, and 360; the remaining seven classes contain 805 GT in total. Only the stated consolidation threshold varies. The frozen candidates, score cutoff, class labels, GT, and greedy class-aware evaluator remain unchanged, including the evaluation mask-IoU threshold of 0.50. Every class row satisfies $TP + FN = GT$ for that class. Both slices recover the same selected-point counts. These are three-class diagnostics, not a complete ten-class or two-dimensional class-wise sweep.

The selected-point sum is $(461, 49, 26) + (97, 12, 6) + (340, 40, 20) + (764, 85, 41) = (1662, 186, 93)$. Reconciliation verifies consistency of the reported summaries; it does not replace candidate hashes or per-object matching traces. The remaining-seven-class totals do not reveal individual category responses. No category-specific operating thresholds are selected from these test results.

Table SXIX. Measured class-wise mask-IoM responses at fixed box-IoU 0.50. The dagger marks the frozen operating value.

Class	Threshold	TP	FP	FN	P	R	F1
Text	0.50	461	48	26	0.9057	0.9466	0.9257
Text	0.60†	461	49	26	0.9039	0.9466	0.9248
Text	0.70	461	49	26	0.9039	0.9466	0.9248
Text	0.80	461	51	26	0.9004	0.9466	0.9229
Text	0.85	462	51	25	0.9006	0.9487	0.9240
Track Section	0.50	95	11	8	0.8962	0.9223	0.9091
Track Section	0.60†	97	12	6	0.8899	0.9417	0.9151
Track Section	0.70	98	12	5	0.8909	0.9515	0.9202
Track Section	0.80	99	13	4	0.8839	0.9612	0.9209
Track Section	0.85	99	13	4	0.8839	0.9612	0.9209
Switch Section	0.50	340	36	20	0.9043	0.9444	0.9239
Switch Section	0.60†	340	40	20	0.8947	0.9444	0.9189
Switch Section	0.70	340	45	20	0.8831	0.9444	0.9128
Switch Section	0.80	343	51	17	0.8706	0.9528	0.9098
Switch Section	0.85	345	59	15	0.8540	0.9583	0.9031

Table SXX. Measured class-wise box-IoU responses at fixed mask-IoM 0.60. The dagger marks the frozen operating value.

Class	Threshold	TP	FP	FN	P	R	F1
Text	0.30	461	48	26	0.9057	0.9466	0.9257
Text	0.40	461	49	26	0.9039	0.9466	0.9248
Text	0.50†	461	49	26	0.9039	0.9466	0.9248
Text	0.60	461	49	26	0.9039	0.9466	0.9248
Text	0.70	461	49	26	0.9039	0.9466	0.9248
Track Section	0.30	97	12	6	0.8899	0.9417	0.9151
Track Section	0.40	97	12	6	0.8899	0.9417	0.9151
Track Section	0.50†	97	12	6	0.8899	0.9417	0.9151
Track Section	0.60	97	12	6	0.8899	0.9417	0.9151
Track Section	0.70	97	12	6	0.8899	0.9417	0.9151
Switch Section	0.30	336	39	24	0.8960	0.9333	0.9143
Switch Section	0.40	337	39	23	0.8963	0.9361	0.9158
Switch Section	0.50†	340	40	20	0.8947	0.9444	0.9189
Switch Section	0.60	341	40	19	0.8950	0.9472	0.9204
Switch Section	0.70	341	41	19	0.8927	0.9472	0.9191

Table SXXI. Count reconciliation. Add the three class rows at the same threshold to the remaining-seven-class counts to obtain the whole-drawing totals. Every total agrees with Table SXII. Other settings are fixed as in the two class tables.

Varied	Value	Other 7 TP	Other 7 FP	Other 7 FN	All 10 TP	All 10 FP	All 10 FN
IoM	0.50	768	115	37	1664	210	91
IoM	0.60	764	85	41	1662	186	93
IoM	0.70	766	126	39	1665	232	90
IoM	0.80	766	147	39	1669	262	86
IoM	0.85	766	161	39	1672	284	83
IoU	0.30	766	102	39	1660	201	95
IoU	0.40	766	108	39	1661	208	94
IoU	0.50	764	85	41	1662	186	93
IoU	0.60	766	111	39	1665	212	90
IoU	0.70	766	112	39	1665	214	90
